# Human Antibodies to VP4 Inhibit Replication of Enteroviruses Across Subgenotypes and Serotypes, and Enhance Host Innate Immunity

**DOI:** 10.3389/fmicb.2020.562768

**Published:** 2020-09-25

**Authors:** Siratcha Phanthong, Jaslan Densumite, Watee Seesuay, Jeeraphong Thanongsaksrikul, Salma Teimoori, Nitat Sookrung, Yong Poovorawan, Napa Onvimala, Ratigorn Guntapong, Kovit Pattanapanyasat, Wanpen Chaicumpa

**Affiliations:** ^1^Graduate Program in Immunology, Department of Immunology, Faculty of Medicine Siriraj Hospital, Mahidol University, Bangkok, Thailand; ^2^Department of Parasitology, Faculty of Medicine Siriraj Hospital, Center of Research Excellence in Therapeutic Proteins and Antibody Engineering, Mahidol University, Bangkok, Thailand; ^3^Graduate Program in Biomedical Science, Faculty of Allied Health Sciences, Thammasat University, Bangkok, Thailand; ^4^Biomedical Research Incubator Unit, Department of Research, Faculty of Medicine Siriraj Hospital, Mahidol University, Bangkok, Thailand; ^5^Department of Pediatrics, Faculty of Medicine, Center of Excellence in Clinical Virology, Chulalongkorn University, Bangkok, Thailand; ^6^Department of Medical Science, Ministry of Public Health, National Institute of Health, Nonthaburi, Thailand

**Keywords:** EV71, coxsackieviruses, VP4, human single-chain antibodies, cell penetrating antibody (transbody), plaque assay, real-time RT-PCR

## Abstract

Hand, foot, and mouth disease (HFMD) is a highly contagious disease that usually affects infants and young children (<5 years). HFMD outbreaks occur frequently in the Asia-Pacific region, and these outbreaks are associated with enormous healthcare and socioeconomic burden. There is currently no specific antiviral agent to treat HFMD and/or the severe complications that are frequently associated with the enterovirus of serotype EV71. Therefore, the development of a broadly effective and safe anti-enterovirus agent is an existential necessity. In this study, human single-chain antibodies (HuscFvs) specific to the EV71-internal capsid protein (VP4) were generated using phage display technology. VP4 specific-HuscFvs were linked to cell penetrating peptides to make them cell penetrable HuscFvs (transbodies), and readily accessible to the intracellular target. The transbodies, as well as the original HuscFvs that were tested, entered the enterovirus-infected cells, bound to intracellular VP4, and inhibited replication of EV71 across subgenotypes A, B, and C, and coxsackieviruses CVA16 and CVA6. The antibodies also enhanced the antiviral response of the virus-infected cells. Computerized simulation, indirect and competitive ELISAs, and experiments on cells infected with EV71 particles to which the VP4 and VP1-N-terminus were surface-exposed (i.e., A-particles that don’t require receptor binding for infection) indicated that the VP4 specific-antibodies inhibit virus replication by interfering with the VP4-N-terminus, which is important for membrane pore formation and virus genome release leading to less production of virus proteins, less infectious virions, and restoration of host innate immunity. The antibodies may inhibit polyprotein/intermediate protein processing and cause sterically strained configurations of the capsid pentamers, which impairs virus morphogenesis. These antibodies should be further investigated for application as a safe and broadly effective HFMD therapy.

## Introduction

Hand, foot, and mouth disease (HFMD) is a highly contagious disease that usually affects infants and children younger than 5 years of age; however, it can occasionally also infect adults (Lee et al., 2009; Kaminska et al., 2013). HFMD outbreaks occur frequently in Asia-Pacific region (Puenpa et al., 2019). The disease is caused by viruses of the family *Picornaviridae*, genus *Enterovirus* – particularly the serotypes coxsackievirus A16 (CVA16) and enterovirus 71 (EV71) (Wu et al., 2010). EV71 is a neurotropic virus, and infection by this virus serotype can lead to severe and life-threatening disease due to neurological, cardiovascular, and respiratory complications, which makes EV71 infection a serious public health threat in endemic countries (Wang et al., 1999; Lin et al., 2003; Cox et al., 2017). There are currently three inactivated EV71 vaccines licensed by the Chinese Food and Drug Administration (Mao et al., 2016; Yi et al., 2017; Qi et al., 2019). These vaccines are effective against EV71 infections, but they have no protective effect against the other HFMD-causing enteroviruses, such as CVA16 and CVA6 (Li J. et al., 2016; Lim and Poh, 2019). To date, there is no direct-acting anti-enterovirus drug to treat HFMD infection or the severe manifestations that often accompany EV71 infection.

EV71 is classified into three genotypes (A, B, and C) and 11 subgenotypes, including A, B1-B5, and C1-C5 (Tee et al., 2010). Like other members of the family *Picornaviridae*, EV71 is a small (30 nm), non-enveloped, positive-sense, single-stranded RNA virus. The icosahedral capsid comprises 60 copies of four structural proteins (VP1, VP2, VP3, and VP4) that are assembled from 12 VP1-VP2-VP3-VP4 pentamers, and each pentamer consists of 5 VP1-VP2-VP3-VP4 protomers. The RNA genome contains one single open reading frame that is flanked by a long 5′-UTR with an internal ribosome entry site and a polyadenylated 3′-UTR. The small VPg protein, which is important for viral replication, is linked covalently to the 5′-UTR (Solomon et al., 2010; Ma et al., 2014). The viral RNA is translated in the cytoplasm into a polyprotein (PP) that is processed by the virus proteases into three precursor proteins (P1, P2, and P3). P1 is cleaved to generate the VP0 (36 kDa), VP1 (33 kDa), and VP3 (27 kDa) structural proteins, which self-assemble into the procapsid (Zhang et al., 2017). The VP0 of the procapsid is further catalyzed into VP2 (28 kDa) and VP4 (8 kDa) to form mature capsid (Jacobson and Baltimore, 1968). Alanine 107 residue on the VP1 (Zhang et al., 2017), and serine 70 residue on the VP2 (Cao et al., 2019) regulate the efficient cleavage of the VP0 precursor into VP2 and VP4 for generating infectious mature virion. The VP1, VP2, and VP3 capsid proteins are surface exposed, while the VP4 lies on the inner surface of the viral procapsid and mature capsid. P2 and P3 are the precursors of seven non-structural proteins, including 2A, 2B, 2C, 3A, 3B (VPg), 3C, and 3D, which are responsible for viral protein processing, genome replication, translation and host factor interaction (Yuan et al., 2018).

At the initial stage of EV71 infection, the virus uses the VP1 ligand to attach to cellular receptors, such as P-selectin glycoprotein ligand-1 (PSGL-1) (Nishimura et al., 2009), scavenger receptor B2 (SCARB2) (Yamayoshi et al., 2009), sialylated glycans (Yang et al., 2009), annexin II (Yang et al., 2011), heparan sulfate (Tan et al., 2013), vimentin (Du et al., 2014), and surface nucleolin protein (Su et al., 2015) – and then the virus particle (∼160S) is endocytosed. Under the acidic pH condition within the endosome, the virus capsid undergoes a conformational change that leads to externalization of the VP4 and the VP1 N-terminus, and then the virus becomes a 135S subviral particle that is called an A-particle (Shingler et al., 2013). The externalized VP1 and VP4 of the A-particle interact with the host endosomal membrane, and the VP4 forms a membrane pore through which the viral RNA exits into the cytoplasm (genome uncoating/release) to initiate viral translation and replication, leaving behind the empty capsid shell (Shingler et al., 2013). Certain cells [e.g., Chinese hamster ovarian (CHO) K1 cells] allow EV71 to attach to the cell membrane, but no infection was observed (Tan et al., 2013). Those authors hypothesized this to be due to failure of the viral genome to enter the cytoplasm. Nevertheless, it was found that the 135S A-particles (with protruded VP4) of poliovirus (also a member of the genus *Enterovirus*; human enterovirus C) could infect CHO-K1 cells and replicate therein (Curry et al., 1996). Mutation of VP4 of poliovirus prevents genome delivery to the cytoplasm (Danthi et al., 2003), which confirms that VP4 is involved in enterovirus genome release. Enterovirus 71 also forms A-particles that works as a gateway to allow genome release (Shingler et al., 2013).

Antibodies have been used for passive immunization against diseases (both post-exposure intervention and disease treatment) even before the discovery of the first antibiotics (Casadevall et al., 2004). Nowadays, infections are commonly treated by using chemical/pharmacologic antimicrobial agents. Nevertheless, antibodies still have therapeutic niche for viral infections, intoxications, envenomation, cancers and inflammatory disorders (Chaisri and Chaicumpa, 2018). Single-chain variable fragment (scFv) or single-chain antibody has emerged as an engineered monovalent antigen-binding molecule. ScFv is a fusion protein of antibody VH and VL domains connected by a short linker peptide (Kulkeaw et al., 2009). The scFv retains the original antibody specificity. Because they are small (∼25–35 kDa), the scFvs have higher tissue penetrating ability than the original four-chain counterpart. They can be produced conveniently *in vitro* by using phage display technology (Smith, 1985; Kulkeaw et al., 2009) or by recombinant antibody technology from a hybridoma or B lymphocyte (Ahmad et al., 2012). Antigen-binding scFvs have been used as therapeutics, diagnostics, and for research (Ahmad et al., 2012). For interfering with the activities of the intracellular proteins such as replicating virus proteins, intracellular toxins, it is necessary that the scFvs are able to access their intracellular target. Therefore, specific scFvs must be developed into a cell penetrable format, i.e., transbodies, by linking the scFvs with cell penetrating peptides (CPPs), such as penetratin (the third helix of *Drosophila* homeodomain) (Derossi et al., 1994), nonaarginine (R9) (Verdurmen et al., 2010; Melikov et al., 2015), which serve as the scFv transduction domain across the otherwise formidable plasma membrane.

VP4 is a highly conserved protein among the EV71 subgenotypes, as well as among other enterovirus A serotypes. Therefore, a drug that targets VP4 should effectuate genome release inhibition, hence replication inhibition of the EV71 across subgenotypes, and possibly across other enterovirus A serotypes. In this study, human single-chain variable antibody fragments (HuscFvs) and their cell penetrable formats (transbodies) specific to EV71-VP4 were generated using phage display technology (Kulkeaw et al., 2009). The HuscFvs and transbodies inhibited replication of EV71 strains of different subgenotypes, as well as other enterovirus serotypes, and enhanced host innate immune response.

## Materials and Methods

### Cells, Viruses, and Virus Propagation

Rhabdomyosarcoma (RD) cells, African green monkey kidney (Vero) cells, and human embryonic kidney (HEK) 293T cells were from American Type Culture Collection (ATCC, Manassas, VA, United States). They were cultured in Dulbecco’s modified Eagle’s medium (DMEM) (Gibco, Thermo Fisher Scientific, Waltham, MA, United States) supplemented with 10% fetal bovine serum (FBS) (HyClone; GE Healthcare Life Sciences, Marlborough, MA, United States), 100 units/mL penicillin, 100 μg/mL streptomycin, and 2 mM L-glutamine (Gibco) (complete DMEM). Chinese hamster ovary K1 (CHO-K1) cells were maintained in RPMI-1640 medium (Gibco) supplemented with the same volumes/amounts of 10% FBS, antibiotics, and glutamine.

EV71 genotype A (BrCr strain) was from ATCC^®^ -VR-1775^TM^; EV71-B5 was isolated from HFMD patients at King Chulalongkorn Memorial Hospital, Bangkok, Thailand; and, EV71-C4 and coxsackieviruses A (CVA), including CVA6 and CVA16, were from the collection of the National Institute of Health, Ministry of Public Health, Nonthaburi, Thailand. The viruses were propagated in either Vero or RD cells grown in complete DMEM at 37°C in a 5% CO_2_ atmosphere. The cytopathic effect (CPE) characterized by cell rounding, clumping, and/or detaching was observed daily. Cells and spent media of individual cultures were harvested when the CPE was clearly observed and pooled. The preparations were subjected to three freeze-thaw cycles, centrifuged to remove the cell debris, and the supernatant containing the virus was kept in small aliquots. The cell culture infectious dose 50 (CCID50) of the viruses were determined (Supplementary Material 1). All virus stocks were maintained at -80°C for later use in virus experiments.

### Production of HuscFvs to EV71-VP4

The HuscFv display phage clones that bound to bacterially derived-recombinant VP4 (rVP4) (Supplementary Material 2) were selected from the phage library, as previously described (Kulkeaw et al., 2009). Briefly, a well of a microplate was coated with 10 μg of purified rVP4. After blocking the plastic surface with 3% BSA, 100 μL of HuscFv phage display library were added into the well and incubated. The unbound phages were removed by washing. The bound phages were used to infect HB2151 *E. coli*, and the preparation was spread onto selective agar plates of 2 × YT containing 100 μg/mL ampicillin and 2% glucose (2 × YT-AG). Phage transformed-HB2151 *E. coli* colonies that appeared on the selective agar plates after overnight incubation at 37°C were screened for the presence of HuscFv genes (*huscfvs*) by direct colony PCR (Kulkeaw et al., 2009). The *huscfv*-positive clones were grown in 2 × YT broth and induced by 0.5 mM isopropyl β-D-1-thiogalactopyranoside (IPTG) (Thermo Fisher Scientific). The expression of E-tagged-HuscFvs in the HB2151 *E. coli* lysates was determined by Western blotting using rabbit anti-E-tag antibody (Abcam, Cambridge, United Kingdom). Binding of the HuscFvs contained in the *E. coli* lysates to rVP4 was checked by indirect ELISA and Western blotting (Kulkeaw et al., 2009).

### Production of Cell Penetrable HuscFvs (Transbodies)

For generation of cell penetrable HuscFvs (transbodies) and large-scale production of transbodies and the original HuscFvs, the *huscfvs* in the pCANTAB5E phagemids (Kulkeaw et al., 2009) were subcloned into pLATE52 expression vector (Thermo Fisher Scientific) with and without DNA sequence coding for cationic cell penetrating peptide (CPP) [i.e., nonaarginine (R9), Melikov et al., 2015] using the ligation independent cloning (LIC) method (aLICator LIC Cloning and Expression Kit 4, Thermo Fisher Scientific). The recombinant plasmids were transformed into JM109 *E. coli*. The *E. coli* clones carrying the recombinant vectors containing *R9-huscfv* or *huscfv* inserts were screened by PCR using LIC primers. The *R9-huscfv/huscfv*-positive clones were cultured, the plasmids were extracted using a plasmid extraction kit (Biotechrabbit, Hennigsdorf, Germany), and then the plasmids were sequenced. The verified plasmids were introduced separately into Rosetta (DE3) *E. coli*. The transformants carrying recombinant *R9-huscfv/huscfvs* plasmids were screened by PCR. Appropriately transformed bacterial colonies were grown individually in 250 mL of 2 × YT broth containing 100 μg/mL ampicillin, 34 μg/mL chloramphenicol, and 1 mM IPTG. The bacterial cultures were maintained at 30°C with shaking at 250 rpm for 6 h. The bacterial inclusion bodies (IBs) containing R9-HuscFvs/HuscFvs were purified and refolded as described previously (Jittavisutthikul et al., 2016). The purity of the recombinant antibodies was determined by sodium dodecyl sulfate-polyacrylamide gel electrophoresis (SDS-PAGE) and Coomassie Brilliant Blue G-250 (CBB) staining. Binding of the R9-HuscFvs/HuscFvs to rVP4 was checked by indirect ELISA and Western blot analysis (Jittavisutthikul et al., 2016).

### Immunofluorescence Assay

Immunofluorescence assay was performed to test cell penetrating ability of the non-R9-HuscFvs and R9-HuscFvs in the non-infected and the EV71 infected-RD cells. For this experiment, RD cells in complete DMEM were seeded onto glass cover slips placed in the wells of a 24-well culture plate (5 × 10^4^ cells/well), and incubated at 37°C in a 5% CO_2_ atmosphere overnight. For preparing infected cells, the RD cell monolayer were rinsed and infected with EV71-B5 at a multiplicity of infection (MOI) of 0.01. After allowing virus entry, the extracellular viruses were removed, the cells were then washed twice and incubated at 37°C overnight. HuscFvs/R9-HuscFvs (25 μg) was added separately to non-infected and infected RD cells and incubated with the cells at 37°C overnight. Culture fluids were then discarded and the cells were rinsed with PBS, fixed, and permeated with cold acetone-methanol mixture (1:1) at −20°C for 20 min. After washing with PBS and blocking with 5% BSA, 300 μL of rabbit anti-E-tag were added to the cells and kept for 1 h. AlexaFlour^®^594-conjugated goat anti-rabbit immunoglobulins (Life Technologies, Carlsbad, CA, United States) (1:200 dilution) were added, followed by DAPI to locate the nuclei. Localization of red-labeled HuscFvs/R9-HuscFvs in the cells were observed under immunofluorescence microscope [40×; LSM800 confocal microscope, Carl Zeiss; image capture program (Zen software version 2)].

For determining co-localization of the intracytoplasmic antibodies with the virally produced VP4, RD cell monolayer were rinsed and infected with EV71-B5 at MOI 0.01. After allowing virus entry, the extracellular viruses were removed. The cells were then washed twice, replenished with complete DMEM, and incubated for 24 h. HuscFvs/R9-HuscFvs (25 μg) was added to incubate with the cells at 37°C overnight. Culture fluids were then discarded and the cells were rinsed with PBS, fixed, and permeated with cold acetone-methanol mixture (1:1) at -20°C for 20 min. After washing with PBS and blocking with 5% BSA, 300 μL of primary antibodies [i.e., mouse anti-VP4 PAb (Supplementary Material 3) (1:200 dilution) and rabbit anti-E-tag antibody (1:3,000 dilution)], and 300 μL of secondary antibodies [i.e., AlexaFlour-488-conjugated donkey anti-mouse antibody (Abcam) (1:200 dilution), and AlexaFlour^®^594-conjugated goat anti-rabbit immunoglobulins (Life Technologies, Carlsbad, CA, United States) (1:200 dilution)] were added sequentially with washing between steps. DAPI (1:5,000 dilution) was used for locating nuclei. The preparations were subjected to sectional confocal microscopy [40×; LSM800 confocal microscope, Carl Zeiss; image capture program (Zen software version 2)] to locate red-labeled HuscFvs/R9-HuscFvs and green-labeled VP4.

### Immunoblotting

Immunoblotting was used in an attempt to determine reactivity (binding) of the HuscFvs/R9-HuscFvs to the enterovirus produced-VP4. Three hundred microliters of 10^7^ CCID_50_/0.1 mL of EV71-B5, EV71-C4 and CVA16 were mixed individually with 60 μL of 6 × SDS Protein Loading Buffer and subjected to SDS-PAGE. The separated components were transblotted onto a nitrocellulose (NC) membrane; the blotted NC strips were blocked with 5% skimmed milk and probed separately with 1 μg of HuscFvs/R9-HuscFvs or control (irrelevant) scFv in Tris-buffered saline containing 0.1% Tween-20 (TBST) or buffer alone. Mouse anti-VP4 PAb (1:1,000 dilution) and anti-VP2 MAb (Chemicon; Merck, Millipore, Burlington, MA, United States) (1:3,000 dilution) were used as positive antibody controls. Following incubation and washing, the membranes were incubated with rabbit anti-E-tag antibody (detected HuscFvs/R9-HuscFvs/control scFv). Alkaline phosphatase (AP)-conjugated anti-isotypic antibody and BCIP/NBT substrate (SeraCare Life Sciences, Milford, MA, United States) were used to visualize the reactive bands.

### Combined Co-immunoprecipitation, ELISA, and Western Blotting

Because the amount of VP4 in the EV71 suspension were too little and we could not see the reactive band of the antibodies and the virally produced-VP4 in the above experiment, therefore we tested binding of the HuscFvs and R9-HuscFvs to VP4 that was overexpressed in HEK 293T cells (Supplementary Material 2). The HEK 293T cell lysate (200 μL) containing Flag-tagged-VP4 was mixed with 2 μg of HuscFvs or R9-HuscFvs in TBST containing 0.1% (w/v) skimmed milk. The mixtures were added to wells of a 96-well plate containing immobilized ANTI-FLAG^®^ M2 (Sigma-Aldrich) and kept at 4°C overnight. After washing, the rabbit anti-E-tag antibody was added. The controls were cell lysate containing overexpressed-VP4 mixed with control (irrelevant) scFv, cell lysate containing overexpressed-VP4 alone, and HuscFvs/R9-HuscFvs mixed with cell lysate without the VP4. After incubation and washing, HRP conjugated-goat anti-isotype was added to each well. ABTS peroxidase substrate was used for color development. The content in each well was measured at absorbance 405 nm (A405 nm) against the blank (PBS). In addition, after reading the plate at A405 nm, the wells were washed, and 6 × protein sample buffer was added to elute the immobilized reactants in each well. The eluates were separated by SDS-PAGE, blotted onto NC membrane, blocked, and probed with anti-Flag-tag (detect Flag-tagged-VP4) and rabbit anti-E-tag. The reactive bands were visualized using BCIP/NBT substrate.

### Biocompatibility of HuscFvs and R9-HuscFvs to Mammalian Cells

Biocompatibility of HuscFvs and R9-HuscFvs with mammalian cells (RD cells as representative) was determined using CellTiter-Glo^®^ Luminescent Cell Viability Assay (Promega). RD cells (5 × 10^5^ cells) in individual wells of a culture plate were added with various amounts (15, 30, 60, and 120 μg) of HuscFvs or R9-HuscFvs in complete DMEM, and incubated overnight. Control was cells in complete DMEM. Working cell viability reagent (provided with the kit) was added to each well. The culture plate was placed on a shaker at 100 rpm for 2 min and kept at room temperature for 10 min. Luminescence of the content in each well was recorded using a Synergy H1 Hybrid Multi-Mode Monochromator Fluorescence Microplate Reader (BioTek Instruments, Winooski, VT, United States). Viability of the cells of each treatment was determined based on the quantity of adenosine triphosphate (ATP), which indicates the presence of metabolically active cells.

### Inhibition of Enterovirus Replication by VP4 Specific-HuscFvs and R9-HuscFvs

To demonstrate that the VP4 specific-antibodies were broadly effective against many subgenotypes of EV71 and enteroviruses that frequently cause HFMD outbreaks, multiple enteroviruses, i.e., EV71 subgenotypes A, B, and C and CVA16 and CVA6, were tested in this experiment. Monolayer of RD cells (2 × 10^5^ cells in 1 mL complete DMEM) established in individual wells of 12-well culture plates were added with enteroviruses using MOI 0.1 for EV71-A (BrCr), EV71-C4 and CVA16; MOI 0.02 for EV71-B5 (this virus is highly virulent for RD cells); and MOI 0.05 for CVA6, and incubated at 37°C for 1 h. Extracellular viruses were discarded by washing. The infected cells were added with 60 μg of HuscFvs or R9-HuscFvs (4 wells for each treatment) and incubated at 37°C in a 5% CO_2_ atmosphere. The culture supernatants and the cells were collected when the CPE caused by individual viruses were clearly seen, i.e., 18 h for CVA16 [CVA16 produces CPE much faster than the EV71 at the same MOI, due to differences in replication kinetics (Lu et al., 2011; Jin et al., 2012) as well as different permissiveness of the RD cells to different enteroviruses (Volle et al., 2019)]; 24 h for EV71-BrCr, EV71-B5 and EV71-C4; and 72 h for CVA6. Controls were virus-infected cells added with 65 μg/mL of ribavirin (nucleoside analog which served as positive virus replication inhibition control), control (irrelevant) antibody (background virus replication inhibition control or placebo), or medium alone (negative virus replication inhibition control). The numbers of infectious viral particles in the culture supernatants of all treatments were determined by plaque assay (Supplementary Material 4). Attached cells were fixed and stained. Some aliquots of the culture supernatants and the attached cells of individual treatments were subjected to RNA extraction using TRIzol^®^ reagent (Ambion; Thermo Fisher Scientific), and the amounts of the virus genomes were quantified by quantitative reverse transcription-PCR (qRT-PCR) (Supplementary Material 5) and compared.

### Peptide-ELISA and Competitive-ELISA for Identification of the VP4 Epitopes Recognized by the Huscfvs and R9-HuscFvs

Twelve biotin-labeled overlapped peptides of the EV71-VP4 (peptides 1–12, started from the first amino acids of the VP4) were synthesized (GenScript). Each peptide contains 15 amino acids with 10 overlapped residues with the adjacent peptide sequences (Supplementary Figure 1). For peptide ELISA, 1 μg of individual peptides in 100 μL carbonate buffer pH 9.6 was used to coat individual wells of a 96-well ELISA plate at 37°C overnight. After washing, the well surface was blocked with 5% skimmed milk. The antibodies to VP4 (100 ng; optimal amount from titration) in TBST were added, incubated at 37°C for 1 h, and washed. Rabbit anti-E-tag antibody and HRP-conjugated goat anti-rabbit isotype were added sequentially with washing between steps. After color development with ABTS [2,2′-azino-bis(3-ethylbenzothiazoline-6-sulfonic acid] substrate, the A450 nm of the content in each well was determined. The peptide that was bound by the antibodies and that gave an optical density (OD_40__5 n__m_) of at least 1.5 times higher than that of the same antibodies to the control peptide (background) was considered to be the presumptive epitope of the antibodies.

For validation of the presumptive epitopic peptides, competitive-ELISA was performed to determine the capacity of the peptides to block binding of the antibodies to the rVP4. The peptides (0.125, 0.25, 0.5, 1, 2, and 4 μg/mL) were mixed with 100 ng of antibodies to VP4, and kept at room temperature for 1 h. Individual mixtures were added to rVP4-coated wells and incubated at 37°C for 1 h. After washing – the rabbit anti-E-tag antibody, HRP-conjugated-goat anti-rabbit isotype, and ABTS peroxidase substrate were added, consecutively, with washing between steps. Antibodies in buffer alone served as negative inhibition controls (maximum binding). The OD_40__5 n__m_ of the content of each well was determined against the blank (TBST). The % ELISA inhibition was calculated, as follows: % ELISA inhibition = 100 × [(OD of maximum binding – OD of test) ÷ (OD of maximum binding)].

### Inhibition of Infectivity of VP4-Exposed EV71 Particles (A-Particles) by VP4 Specific-Antibodies

VP4 of enteroviruses is known to form membrane pore to release virus RNA from endosome into cytoplasm for further replication. Chinese hamster ovarian K1 (CHO-K1) cells have been shown to be non-permissive to wild type EV71 due to the lack of human SCARB2 (Chen et al., 2012; Tan et al., 2013) which is receptor for virus attachment and genome uncoating in endosome (Kobayashi and Koike, 2020), but VP4-exposed EV71 particles (A-particles with protruded VP4) could infect and replicate inside these cells due to the plasma membrane pore-forming activity of the exposed VP4 that enables direct release of the virus genome into the cytoplasm, regardless of the virus-host receptor interaction (Curry et al., 1996; Tan et al., 2013). Therefore, in order to investigate whether the VP4 specific-antibodies could inhibit the virus genome release through interfering with the VP4 membrane activity, the experiments involving CHO-K1 cells infected with A-particles and wild type EV71 were performed and compared. In this experiment, the A-particles were prepared by converting wild type EV71 particles to A-particles *in vitro* using the method described previously (Wetz and Kucinski, 1991) with some modifications. EV71 virus stock was diluted in 20 mM Tris (pH 7.5), 2 mM CaCl_2_, and 0.1% Tween-20, and then incubated at 50°C for 3 min. After incubation, the preparation was mixed with equal volumes (2 mL) of PBS containing 0.5 mM MgCl_2_, 0.7 mM CaCl_2_, and 0.5% (v/v) FBS.

To verify the infectivity of the EV71 A-particles, a CHO-K1 cell monolayer was established in two six-well culture plates (Supplementary Figure 2A). Cells in 3 wells of each plate were added with A-particles or wild type EV71. Immediately after adding the viruses, the fluids in all wells of plate 1 were discarded. The cells were then washed and added with 1 mL of medium followed by TRIzol^®^ reagent for RNA preparation (0 h samples were obtained). Plate 2 was incubated at 37°C in a CO_2_ incubator for 1 h to allow virus entry. Thereafter, fluids in all wells were removed and the cells were washed, replenished with 1 mL of fresh culture medium, and incubated further for 5 h. After incubation (6 h post-infection), all wells were added with TRIzol^®^ reagent (6 h samples were obtained). Virus RNA amounts in the 0 and 6 h samples were quantified by qRT-PCR.

In order to determine the ability of the VP4 specific-antibodies to inhibit A-particle infectivity *via* inhibition of membrane pore-forming activity of VP4, A-particles in medium, mixtures of A-particles and HuscFvs/R9-HuscFvs/control (irrelevant) scFv, or wild type EV71 in medium were added individually to 3 wells containing 10^6^ CHO-K1 cells in 6-well culture plates (Supplementary Figure 2B). The fluids in all wells of plate 1 were discarded immediately. The wells were then washed, added with 1 mL of fresh culture medium, and the plate was subjected to three freeze-thaw cycles. The cell debris was removed by centrifugation and the 0 h samples were obtained. The remaining plates were incubated for 1 h, the fluids in all wells were discarded, and the cells were washed, added with 1 mL of fresh culture medium, and incubated further for 5 h. The plates were then subjected to three freeze-thaw cycles, the cell debris in all wells was removed by centrifugation, and the 6 h samples were obtained. The infectious virus titers in the 0 and 6 h samples were determined by plaque assay performed on the RD cells, and the results were compared among all treatments.

### Effect of VP4 Specific-Antibodies on Capsid Protein Production

In the case that the VP4 specific-antibodies interfered with the genome release activity of the VP4, this should lead to not only less virus genome replication, but also reduced production of intracellular virus proteins. Experiment to test this perspective was carried-out. RD cells (1.7 × 10^6^ cells in 5 mL complete DMEM) in T25 culture flasks were infected with 100 μL of 10^6^ CCID50 of EV71 in 2 mL of plain DMEM (MOI 1.0) at 37°C for 1 h. The unbound viruses were then removed by discarding the fluids in the flasks and rinsing the cells twice with PBS. After that, HuscFvs/R9-HuscFvs (50, 100, and 200 μg or 15, 30 and 60 μg per 5 × 10^5^ RD cells – the doses which were not cytotoxic) in 5 mL of plain DMEM containing antibiotic and L-glutamine were added with subsequent incubation at 37°C in a 5% CO_2_ atmosphere overnight. Controls included infected cells added with control scFv antibody, infected cells without antibodies, and uninfected (normal) cells in culture medium. After incubation, the content in each flask was subjected to three freeze-thaw cycles, and the debris was removed by centrifugation. Each lysate was precipitated by mixing with 1:4:4 of chloroform:methanol:sample and centrifugation at 13,000 × g for 5 min. The pellets were washed with methanol, dried, and added with 50 μL of protein sample buffer before subjecting to Western blot analysis by probing the SDS-PAGE-separated components with mouse anti-VP2 MAb (1:3,000) and mouse anti-β-actin MAb (1:5,000). HRP-conjugated mouse IgGκ binding protein (Santa Cruz Biotechnology, Dallas, TX, United States) (1:5,000) and substrate were added, consecutively, with washing between steps. The relative amounts of VP0 and VP2 (representatives of viral capsid proteins), and β-actin were visualized by enhanced chemiluminescence (ECL).

### Determination of Innate Immune Response of Enterovirus-Infected Cells After Treatment With HuscFvs and R9-HuscFvs

Inhibition of the virus replication should also result in less production of the virus proteins not only the capsid (structural) proteins, but also non-structural proteins including 2A, 2C, 3C, and 3D which have been known to inhibit the anti-viral innate immunity (please see Discussion). Therefore, experiments were set up to determine whether the VP4 specific-antibodies could enhance (restore) innate immunity of the enterovirus-infected cells. For determination of the innate immune response of the virus-infected cells after treatment with HuscFvs/R9-HuscFvs, RD cells infected with EV71-B5 (representative) at an MOI of 0.1 were incubated at 37°C in a 5% CO_2_ atmosphere with 60 μg of antibodies, medium alone (negative replication inhibition control), or 65 μg/mL ribavirin (positive replication inhibition control) for 6, 12, 24, and 36 h. RD cells treated with 1 μg/mL of high molecular weight polyinosinic-polycytidylic acid [poly (I:C) HMW] (InvivoGen, Pak Shek Kok, Hong Kong) were used as positive gene induction control. The total RNA of cells from individual treatments was extracted, and 200 ng of each RNA preparation were subjected to qRT-PCR. The ΔCt values of individual innate immune response genes, including *IFN*-α, *IFN*-β, and *ISGs* (*OAS*, *MxA*, and *PKR*), were compared with the housekeeping gene (ribosomal protein lateral stalk subunit P0, RPLP0) and subtracted by background ΔCt of normal cells. Data (ΔΔCt) are expressed as fold changes of individual genes.

### Statistical Analysis

Experimental results of different treatments are shown as mean ± SD. GraphPad Prism version 6 (GraphPad Software, Inc., San Diego, CA, United States) was used for statistical analysis by one-way analysis of variance (ANOVA) or two-way ANOVA. A *p*-value less than 0.05 was considered statistically significant.

## Results

### Recombinant VP4 (rVP4) and HuscFvs to rVP4

**FIGURE 1:**
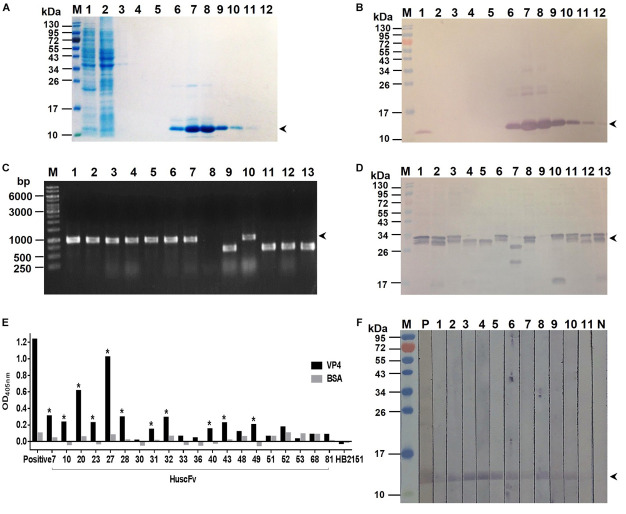
Production of recombinant (r) VP4 and HuscFvs that bound to rVP4. **(A)** SDS-PAGE-separated rVP4 preparation stained by Coomassie Brilliant Blue (CBB) dye. Lane 1, proteins in whole cell lysate of *E. coli* carrying recombinant pET-23b(+) vector with inserted cDNA coding for full-length VP4; lane 2, proteins in *E. coli* lysate that did not bind to the anti-6 × -His column (flow-through); lanes 3–5, column-washed fractions 1–3, respectively, lanes 6–12, column-eluted fractions containing purified rVP4 (arrowhead). **(B)** Western blot patterns of SDS-PAGE-separated rVP4 preparations as in **(A)** probed with anti-6 × His antibody. Arrowhead indicates the rVP4-anti-6× His reactive bands. **(C)** PCR amplicons of HuscFv genes *(huscfvs*) of about 1,000 base pairs (bp) (arrowhead) from representative phage-transformed *E. coli* clones. **(D)** HuscFvs expressed from representative *huscfv*-positive *E. coli* clones (bands of protein doublet at about 26–34 kDa; arrowhead). **(E)** Binding of HuscFvs in lysates of *E. coli* clones to rVP4 as determined by indirect ELISA using BSA as control antigen and lysate of original HB2151 *E. coli* as negative antibody control. Eleven *E. coli* clones (clones 7, 10, 20, 23, 27, 28, 31, 32, 40, 43, and 49, asterisks) expressed HuscFvs that gave significant ELISA OD_405_ nm above controls; Positive, anti-6 × His antibody bound to 6 × His-tagged-rVP4. **(F)** HuscFvs in lysates of the 11 *E. coli* clones bound to SDS-PGE separated-rVP4 (bands at ∼12 kDa in lanes 1–11) as determined by Western blot analysis; P, positive control (i.e., SDS-PAGE separated-rVP4 probed with anti-6 × His antibody); N, negative control (i.e., SDS-PAGE separated-rVP4 probed with lysate of original HB2151 *E. coli*). Lanes M of **(A,B,D,F)** are protein molecular mass markers. Numbers at the left of **(A,B,D,F)** are protein masses in kDa. The numbers to the left of **(C)** are DNA sizes in bp.

Bacterially derived-recombinant EV71-VP4 (rVP4) was produced for use as antigen to select out the rVP4-bound phage clones from the HuscFv-phage display library by panning process (Kulkeaw et al., 2009). VP4 was produced and purified from lysate of transformed *E. coli* bacteria carrying pET-23b(+) vector with inserted full-length EV71-VP4 cDNA. The recombinant protein was checked by SDS-PAGE and protein staining (Figure 1A), Western blot analysis (Figure 1B), and verified as EV71-VP4 by mass spectrometry (data not shown). The purified rVP4 was used as an antigen for selecting phage clones that displayed the rVP4-bound HuscFvs from a previously constructed HuscFv phage display library. *E. coli* HB2151 infected with rVP4-bound phages were screened by PCR for the presence of genes coding for HuscFvs (*huscfvs*, ∼1,000 bp), and 44 phage-transformed *E. coli* colonies were positive for the *huscfvs* (Figure 1C). Among them, 20 clones expressed soluble HuscFvs (Figure 1D), and the HuscFvs of 11 *E. coli* clones (numbers 7, 10, 20, 23, 27, 28, 31, 32, 40, 43, and 49) bound to rVP4 by indirect ELISA (Figure 1E) and Western blot analysis (Figure 1F). The amino acid sequences of the HuscFvs of the 11 clones could be classified into 8 groups, as follows: group 1 (clones 7 and 28), group 2 (clones 32 and 40), group 3 (clones 23 and 49), and groups 4–8 were clones 10, 20, 27, 31, and 43, respectively. The *huscfvs* of the selected HB2151 *E. coli* clones were subcloned from phagemids to protein expression plasmids and the plasmids were used to transfect Rosetta (DE3) *E. coli* for production of cell penetrating peptide (R9)-linked-HuscFvs in large scale.

### Preparation and Purification of VP4 Specific- HuscFvs and R9-HuscFvs and Their Biocompatibility to Mammalian Cells

**FIGURE 2:**
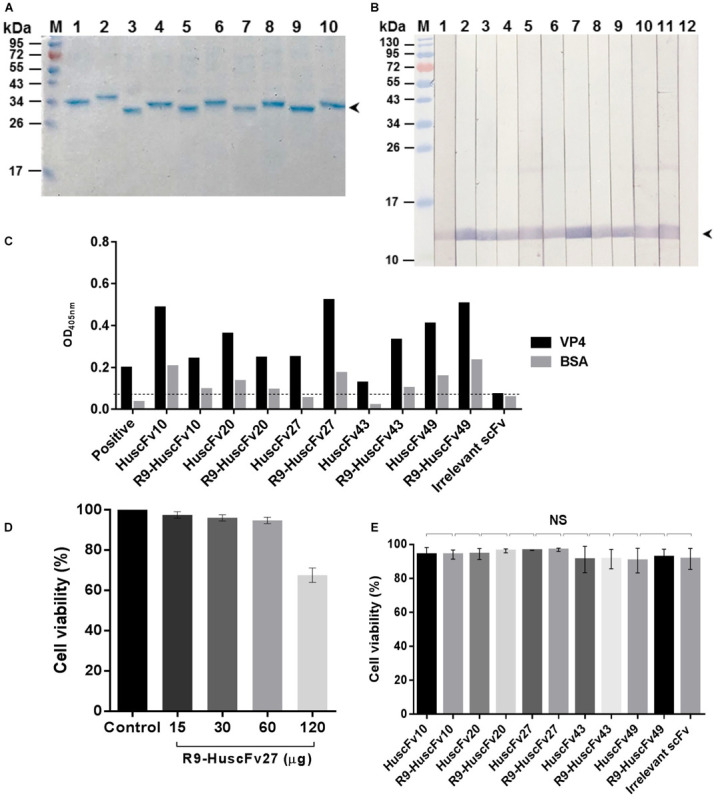
Purified HuscFvs and R9-HuscFvs, and their binding to rVP4 and biocompatibility to mammalian cells. **(A)** Purified HuscFvs (lanes 1, 3, 5, 7, and 9) and their respective R9-HuscFvs (lanes 2, 4, 6, 8, and 10) from the transformed *E. coli* clones 10, 20, 27, 43, and 49, respectively, as determined by SDS-PAGE and CBB staining. **(B)** Binding of HuscFvs (lanes 2, 4, 6, 8, and 10) and their respective R9-HuscFvs (lanes 3, 5, 7, 9, and 11) to SDS-PAGE-separated rVP4 (arrowhead) as determined by Western blot analysis using control (irrelevant) scFv for negative binding (lane 12) and anti-6 × His antibody for positive binding (lane 1). **(C)** Binding of HuscFvs and their respective R9-HuscFvs to rVP4 as determined by indirect ELISA using BSA as control antigen and control (irrelevant) scFv. Positive, recombinant VP4-coated well added with anti-6 × His antibody. **(D)** Percent viability of the RD cells after incubation overnight with various amounts of R9-HuscFv27 (representative) compared with the cells in medium alone (control). **(E)** Viability of RD cells incubated with 60 μg of HuscFvs, R9-HuscFvs, and control antibody.

R9-HuscFvs/HuscFvs expressed by the plasmid-transformed Rosetta (DE3) *E. coli* clones 10, 20, 27, 43, and 49 were purified (Figure 2A). The purified R9-HuscFvs still bound to the rVP4 similar to the behavior of their original HuscFvs (Figures 2B,C). Fifteen, thirty, and sixty micrograms of antibodies incubated overnight with RD cells revealed negligible cytotoxicity (representative is shown in Figure 2D). The results in Figure 2E indicate that the HuscFvs and R9-HuscFvs at 60 μg did not affect the viability of the mammalian cells.

The preliminary results of EV71-neutralization assays indicated that R9-HuscFv27, R9-HuscFv43, R9-HuscFv49 and the original HuscFvs27, HuscFv43 and HuscFv49 were effective in inhibiting replication of the EV71. However, after subcloning, the HuscFv43 and HuscFv49 were not expressed adequately; therefore, only the R9-HuscFv27, R9-HuscFv43, R9-HuscFv49, and HuscFv27 were studied further.

### Cell Penetrating Ability of HuscFvs and R9-HuscFvs and Their Co-localization With Virally Produced-VP4 and Binding to HEK Overexpressed-VP4

**FIGURE 3:**
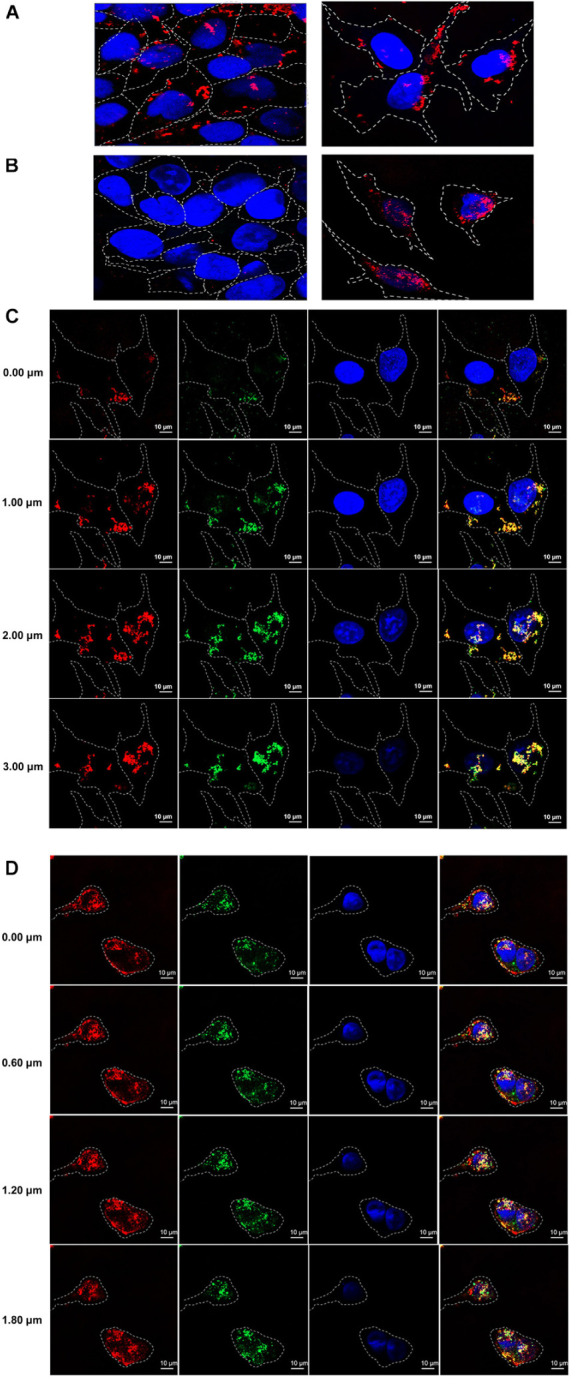
Cell penetrating ability of R9-HuscFvs and HuscFvs. **(A)** R9-HuscFvs (R9-HuscFv27 representative) entered non-infected RD and EV71-infected cells (red in cytoplasm; some located at nuclei which appear blue from DAPI staining). **(B)** Non-R9 HuscFv27 could not enter the non-infected RD cells (left panel), but was able to effortlessly enter the EV71-infected cells; nuclei stained blue. **(C)** R9-HuscFvs (R9-HuscFv27 representative) entered the EV71-infected cells (red in the column 1); the virally produced-VP4 appears green in column 2; nuclei stained blue by DAPI in columns 3 and 4. R9-HuscFv27 co-localized with the virally produced-VP4 and appears in orange or yellow in column 4, which is a merged representation of columns 1, 2, and 3. **(D)** Non-R9 HuscFv27 could enter EV71-infected cells (red in column 1) and also co-localized with intracellular VP4 (green in column 2), which appears orange or yellow in column 4 (merged columns 1, 2, and 3). Nuclei appear blue by DAPI staining in columns 3 and 4.

In order to test cell penetrating ability of the HuscFvs/R9-HuscFvs, immunofluorescence assay was performed. The R9-HuscFv27 (representative) could enter both non-infected and infected RD cells (appeared red in both panels of Figure 3A). The non-R9-HuscFv27 could not enter the non-infected RD cells but readily enter the infected RD cells (appear red in the right panel of Figure 3B).

Sectional confocal microscopy was performed to demonstrate co-localization of the HuscFvs/R9-HuscFvs with the cytoplasmic VP4 (which could be in the nascent polyprotein PP, intermediate P1 or intermediate VP0 protein). Figure 3C shows that the R9-linked-HuscFvs (R9-HuscFv27 as representative) not only enter the EV71-infected cells (red in column 1), but also co-localized with the VP4 (green in column 2) which the co-localized-R9-HuscFv27 and the virally produced-VP4 appeared orange or yellow in column 4 (a merged representation of columns 1, 2, and 3). Likewise, Figure 3D demonstrated that the non-R9-HuscFv27 not only could enter the EV71-infected cells (red in column 1), but also co-localized with intracellular VP4 which appears orange or yellow in column 4 (merged representation of columns 1, 2, and 3). Nuclei appear blue by DAPI staining in columns 3 and 4 of Figures 3C,D.

**FIGURE 4:**
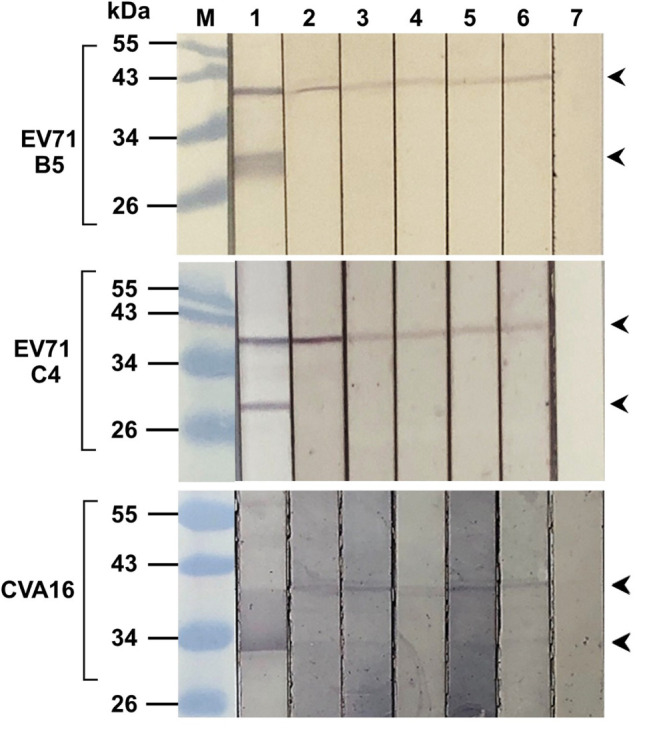
Binding of R9-HuscFv27, R9-HuscFv43, R9-HuscFv49, and HuscFv27 to capsid proteins produced by enterovirus. SDS-PAGE-separated lysates of EV71-B5 (upper block), EV71-C4 (middle block), and CVA16 (lower block) were probed with tested antibodies to VP4 [i.e., R9-HuscFv27, R9-HuscFv43, R9-HuscFv49, and HuscFv27 (lanes 3–6, respectively)] and control scFv (lane 7); lanes 1 and 2, the respective virus lysates probed with anti-VP2 MAb and anti-VP4 PAb, respectively, which served as positive reaction controls. Anti-VP2 MAb reacted to VP0 (precursor of VP2 and VP4) (upper arrowheads) and VP2 (lower arrowheads). All of the tested antibodies and anti-VP4 PAb reacted to VP0 only, while the control (irrelevant) antibody did not reveal any reactive band to the virus proteins. Lane M, protein molecular weight markers. The numbers to the left of all blocks are protein molecular masses in kDa.

From Figures 3C,D, the intracellular antibodies co-localized specifically with the VP4; therefore, attempt to demonstrate binding of the antibodies to the virally produced-VP4 was performed by immunoblotting. Enterovirus stock was lysed and subjected to SDS-PAGE, blotted onto nitrocellulose membrane (NC), and probed with R9-HuscFv27, R9-HuscFv43, R9-HuscFv49, and HuscFv27. Mouse anti-VP4 polyclonal antibody (PAb) and anti-VP2 monoclonal antibody (MAb) were used as positive binding controls. Control (irrelevant) scFv served as negative antibody. Figure 4 shows that the anti-VP4 PAb and the R9-HuscFv27, R9-HuscFv43, R9-HuscFv49, and HuscFv27 bound to VP0 (precursor of VP2 and VP4) revealed by reactive bands at ∼36 kDa (lanes 2–6, respectively). The anti-VP2 MAb revealed reactive bands at ∼36 kDa (location of VP0) and ∼28 kDa (location of VP2) (lane 1). The control (irrelevant) antibody yielded no reactive band. The results indicate that the R9-HuscFv27, R9-HuscFv43, R9-HuscFv49, and HuscFv27 that bound to bacterially produced-VP4 also bound to the EV71 produced-VP4 in the VP0 polyprotein.

**FIGURE 5:**
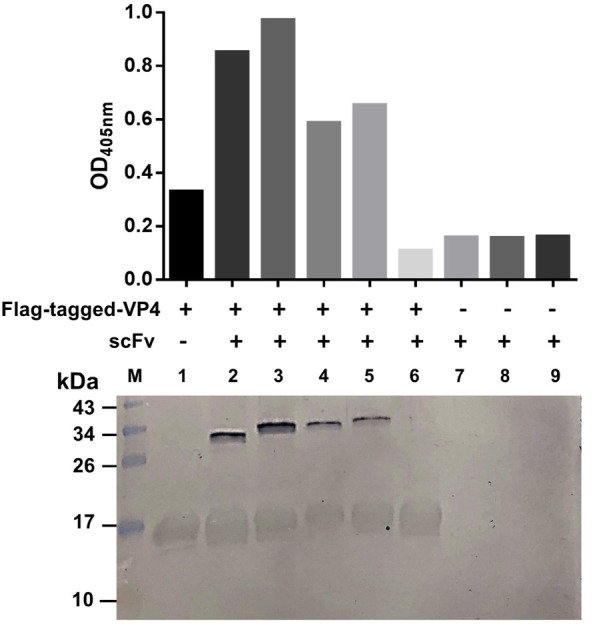
Binding of R9-HuscFvs27, R9-HuscFvs43, R9-HuscFvs49, and HuscFvs27 (tested antibodies) to mammalian cell overexpressed-VP4. Bar graphs demonstrate ELISA OD_40__5 n__m_ of the interaction between the overexpressed-VP4 and the tested antibodies. In this experiment, the lysate of pCI-neo vector containing Flag-tag DNA-*VP4* transfected-HEK 293T containing VP4 was mixed with the tested and control antibodies, and the individual mixtures were added to ELISA wells containing immobilized anti-Flag antibody (wells 2–6, respectively). Well 1 contained the immobilized anti-Flag added with the cell lysate containing the Flag-tagged-VP4, and wells 7–9 contained R9-HuscFv27, R9-HuscFv43, and R9-HuscFV49 alone (background controls). All of the tested antibodies bound to Flag-tagged-VP4 and gave high ELISA signals (bars 2–5) compared to the control antibody mixed with cell lysate containing Flag-tagged-VP4 (bar 6), and the background controls (bars 7–9). Lower panel shows Western blot patterns of the eluted substances from the respective ELISA wells after the fluids in all wells were discarded. Both Flag-tagged-VP4 (∼17 kDa) and R9-HuscFvs (∼34–40 kDa) were detected in wells added with mixtures of the cell lysates containing Flag-tagged-VP4 and the tested antibodies (lanes 2–5), which indicates that the tested antibodies bound to the VP4 and were detected together in the Flag-tagged-VP4-HuscFv complexes captured by the immobilized anti-Flag antibody. Lane 1 contained only the VP4. Lanes 7–9 did not have the VP4, thus the added antibodies were not bound by the anti-Flag. Lane M, protein molecular weight markers (kDa). The numbers to the left of the lower panel are protein molecular masses in kDa.

Because the VP4 in the virus lysate on the blotted NC was too little to reveal a reactive band with the antibodies in the above experiment, we then performed the experiment to test the binding of the R9-HuscFv27, R9-HuscFv43, and R9-HuscFv49 and HuscFv27 to HEK overexpressed-VP4. The antibodies to VP4 were mixed individually with HEK cell lysate containing the overexpressed/Flag-tagged-VP4, and the individual mixtures were added to anti-Flag-tag immobilized in microplate wells. Rabbit anti-E-tag was used to detect the R9-HuscFvs/HuscFv27, and ELISA reagents were added as needed to complete the reaction. The three transbodies and the HuscFv27 gave high ELISA signals to the overexpressed-VP4 in the HEK cell lysate compared to controls (antibodies without HEK cell lysate containing the VP4) (Figure 5, bar graphs). Thereafter, the fluids in all ELISA wells were discarded and the wells were washed. The immobilized complexes were eluted out by adding small volumes of protein sample buffer and subjecting them to SDS-PAGE and Western blotting by probing with anti-Flag-tag and anti-E-tag antibodies. Lower panel of Figure 5 shows that the Flag-tagged-VP4 and the respective E-tagged R9-HuscFvs/HuscFv27 were found in the same elutes (lanes 2–5), which indicates that they were interacted within the eluted complexes.

Taken together, the overall results indicated that the VP4 specific-antibodies not only co-localized with the intracellular VP4 in the infected cells, but also could bind to the virally produced-VP0, which is the intermediate protein (precursor of VP4 and VP2) for the morphogenesis of the virus progeny, as well as the overexpressed-VP4 in mammalian cells.

### Inhibition of Enterovirus Replication by the Antibodies to VP4

**FIGURE 6:**
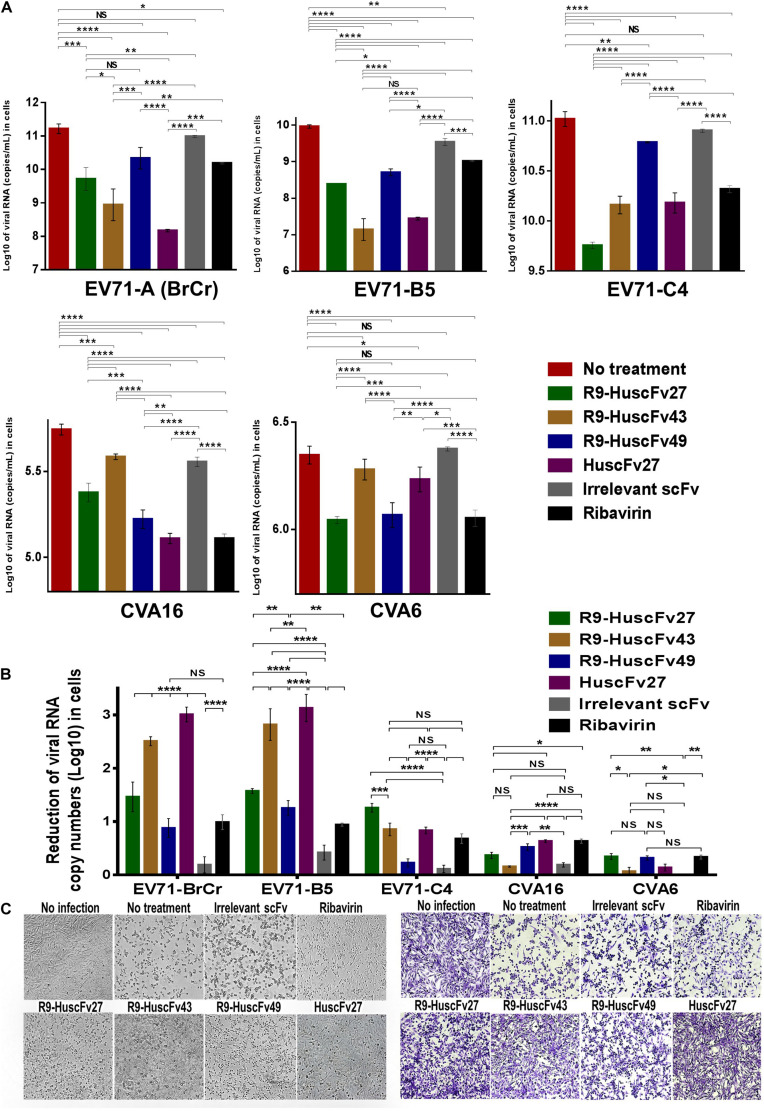
Inhibition of enterovirus replication by VP4 specific-antibodies. **(A)** Log_10_ of viral RNA in RD cells infected with enteroviruses EV71-A (BrCr), EV71-B5, EV71-C4, CVA16, and CVA6 that were treated with medium alone, R9-HuscFv27, R9-HuscFv43, R9-HuscFv49, HuscFv27, control (irrelevant) scFv, or ribavirin. **(B)** Reduction in viral RNA copy numbers (log_10_) in enteroviruses infected RD cells after treatments with VP4 specific-antibodies and controls (irrelevant scFv and ribavirin) compared to respective enteroviruses infected cells without treatment (infected cells in medium alone) as determined by qRT-PCR. **(C)** Normal RD cell monolayer and representative CPE of EV71 infected RD cells without treatment and treatment with irrelevant (control) antibody, ribavirin, R9-HuscFv27, R9-HuscFv43, R9-HuscFv49, and HuscFv27 before (left panel group) and after (right panel group) staining. *, *p* < 0.05; **, *p* < 0.01; ***, *p* < 0.001; ****, *p* < 0.0001.

**FIGURE 7:**
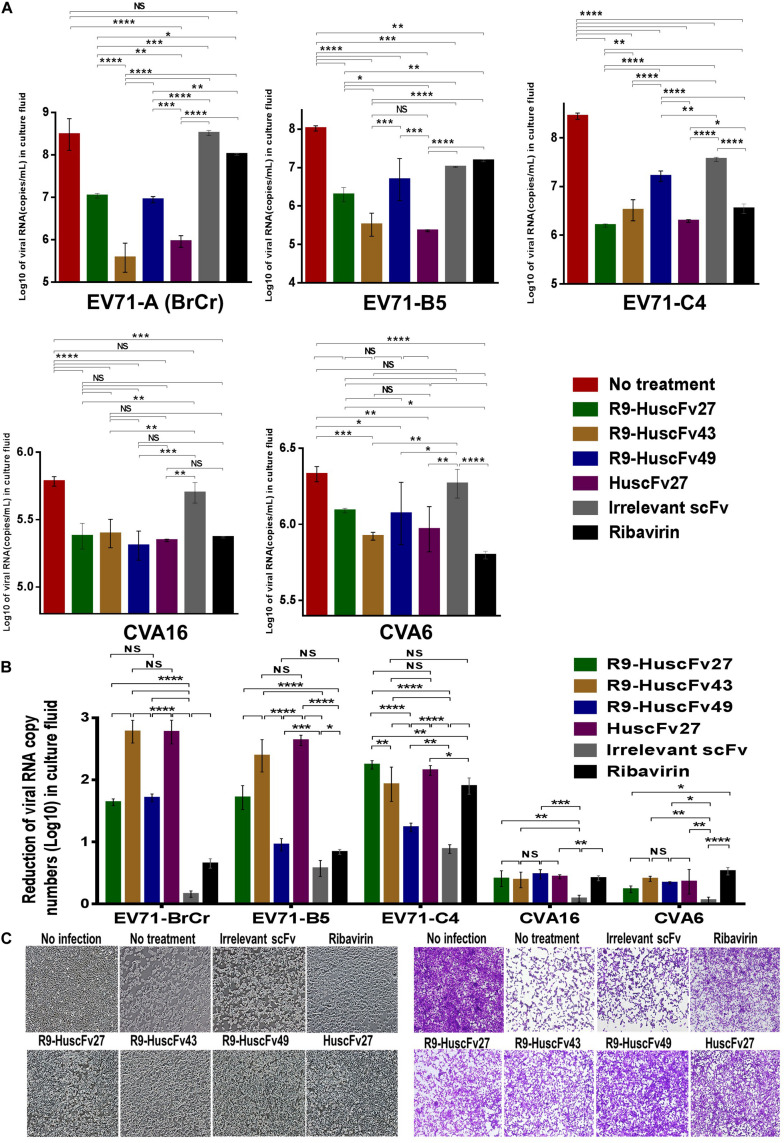
Inhibition of enterovirus replication by VP4 specific-antibodies. **(A)** Log_10_ of viral RNA in culture supernatants of RD cells infected with enteroviruses EV71-A (BrCr), EV71-B5, EV71-C4, CVA16, and CVA6 that were treated with medium alone, R9-HuscFv27, R9-HuscFv43, R9-HuscFv49, HuscFv27, control (irrelevant) scFv, or ribavirin. **(B)** Reduction in viral RNA copy numbers (log_10_) in culture supernatants of enteroviruses infected RD cells after treatments with VP4 specific-antibodies and controls (irrelevant scFv and ribavirin) compared to respective enteroviruses infected cells without treatment (infected cells in medium alone) as determined by qRT-PCR. **(C)** Normal RD cell monolayer and representative CPE of CVA16 infected RD cells without treatment and treatment with irrelevant (control) antibody, ribavirin, R9-HuscFv27, R9-HuscFv43, R9-HuscFv49, and HuscFv27 before (left panel group) and after (right panel group) staining. *, *p* < 0.05; **, *p* < 0.01; ***, *p* < 0.001; ****, *p* < 0.0001.

Therapeutic ability of the VP4 specific-antibodies in inhibiting replication of enteroviruses that frequently cause epidemics (EV71-A, EV71-B, EV71-C, CVA16, and CVA6) were determined. RD cells infected with enteroviruses EV71-A (BrCr), EV71-B5, EV71-C4, CVA16, and CVA6 for 1 h were treated with R9-HuscFv27, R9-HuscFv43, R9-HuscFv49, HuscFv27, ribavirin (nucleoside analog; positive replication inhibition control), medium alone (negative replication inhibition control), or control (irrelevant) antibody (background replication inhibition control or placebo). All of the antibodies to VP4 and ribavirin could inhibit replication of the enteroviruses that were tested, compared to negative and background replication inhibition controls for individual viruses, as indicated by reduction of the RNA copy numbers of the respective viruses inside the infected cells (Figure 6A) and in the respective culture supernatants (Figure 7A). The effectiveness of different antibodies compared to controls (ribavirin and control antibody) on each studied enterovirus (shown as log_10_ reduction of the viral RNA copy numbers in infected RD cells and in culture supernatants, are summarized in Figures 6B, 7B, respectively. Representative CPE of EV71-BrCr- and CVA16- infected cells treated with different antibodies and controls before and after staining, are shown in Figures 6C, 7C, respectively.

The comparative extent of the effectiveness of the antibodies and ribavirin against the viruses is shown in Table 1. Some of VP4 specific-antibodies were more effective than the ribavirin especially for EV71 subgenotypes. The comparative results of plaque assay for detecting infectious virus particles (PFU/mL) in the culture supernatants of infected RD cells after various treatments shown in Supplementary Figure 3 were in concordance with the results of log_10_ reduction of the viral RNA copy numbers in the respective culture supernatants. The HuscFv27/R9-HuscFv27 seemed to be more effective than the other two antibodies in the virus replication inhibition.

**TABLE 1:** Effectiveness of VP4 specific-antibodies and controls in reducing the amount of viral RNA of various enteroviruses.

Enterovirus	Culture fraction of infected RD cells	Effectiveness of the VP4 specific-antibodies in reducing the amount of viral RNA
EV71-A (BrCr)	Cells	HuscFv27 > R9-HuscFv43 > R9-HuscFv27 > R9-HuscFv49 = ribavirin > irrelevant scFv
	Culture fluid	HuscFv27 = R9-HuscFv43 > R9-HuscFv27 = R9-HuscFv49 > ribavirin > irrelevant scFv
EV71-B5	Cells	HuscFv27 > R9-HuscFv43 > R9-HuscFv27 > R9-HuscFv49 > ribavirin > irrelevant scFv
	Culture fluid	HuscFv27 = R9-HuscFv43 > R9-HuscFv27 > R9-HuscFv49 = ribavirin > irrelevant scFv
EV71-C4	Cells	R9-HuscFv27 > HuscFv27 = R9-HuscFv43 = ribavirin > R9-HuscFv49 = irrelevant scFv
	Culture fluid	HuscFv27 = R9-HuscFv27 > R9-HuscFv43 = ribavirin > R9-HuscFv49 > irrelevant scFv
CVA16	Cells	HuscFv27 = ribavirin = R9-HuscFv49 > R9-HuscFv27 > R9-HuscFv43 = irrelevant scFv
	Culture fluid	HuscFv27 = ribavirin = R9-HuscFv49 = R9-HuscFv27 = R9-HuscFv43 > irrelevant scFv
CVA6	Cells	R9-HuscFv27 = R9-HuscFv49 = ribavirin > HuscFv27 > R9-HuscFv43 > irrelevant scFv
	Culture fluid	HuscFv27 = R9-HuscFv49 = ribavirin = R9-HuscFv43 > R9-HuscFv27 > irrelevant scFv

### Determination of the VP4 Regions (Epitopes) Bound by the Antibodies

**FIGURE 8:**
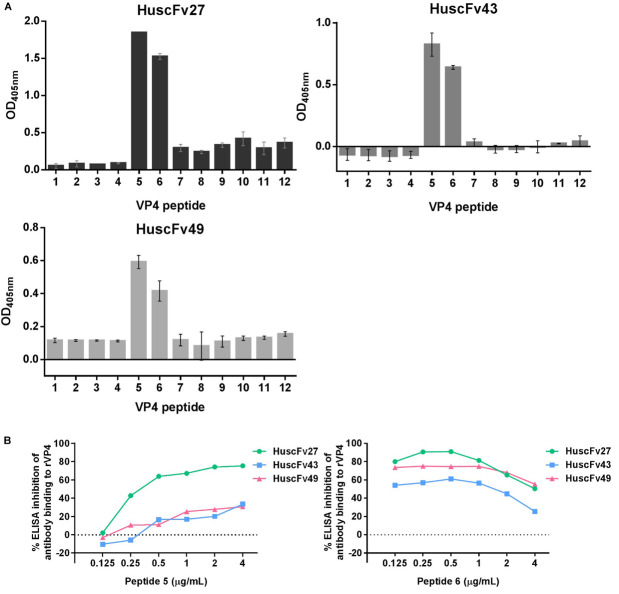
Determination of VP4 peptides bound by HuscFv27, HuscFv43, and HuscFv49 by indirect ELISA, and verification of the epitopic peptides by competitive ELISA. **(A)** Binding of HuscFv27, HuscFv43, and HuscFv49 to the 12 biotin-labeled overlapped peptides of EV71-VP4 (peptides 1–12) as determined by indirect ELISA. All of the tested VP4 specific-antibodies bound mainly to peptides 5 and 6. **(B)** Results of competitive ELISA using various concentrations of peptides 5 and 6 (0.125, 0.25, 0.5, 1, 2, and 4 μg/mL) to mix with HuscFv27, HuscFv43, and HuscFv49 before adding to the rVP4-coated wells. Both VP4 peptides could inhibit the binding of their respective antibodies to immobilized rVP4 in a dose-dependent manner (0.125, 0.25, and 0.5 μg/mL). Inhibition was reduced in the zone of antigen excess. The results indicate that the antibodies bound to the target VP4 at the overlapped region of peptides 5 and 6.

For determining the location of the VP4 bound by the effective antibodies, 12 biotin-labeled overlapped peptides of the EV71-VP4 (Supplementary Figure 1) were immobilized separately in wells of a microplate. The antibodies to VP4 were added, and the antibodies that bound to the immobilized peptides were detected by ELISA. Figure 8A shows that all of the VP4 specific-antibodies bound mainly to peptides 5 and 6. Both peptides could inhibit binding of the antibodies to the immobilized rVP4 in a peptide dose-dependent manner (Figure 8B), which indicates that the antibodies bound to VP4 peptides 5 and 6 overlapped region, residues 25NYTTINYYKD34, which was regarded as the VP4 N-terminal portion (Panjwani et al., 2016).

**FIGURE 9:**
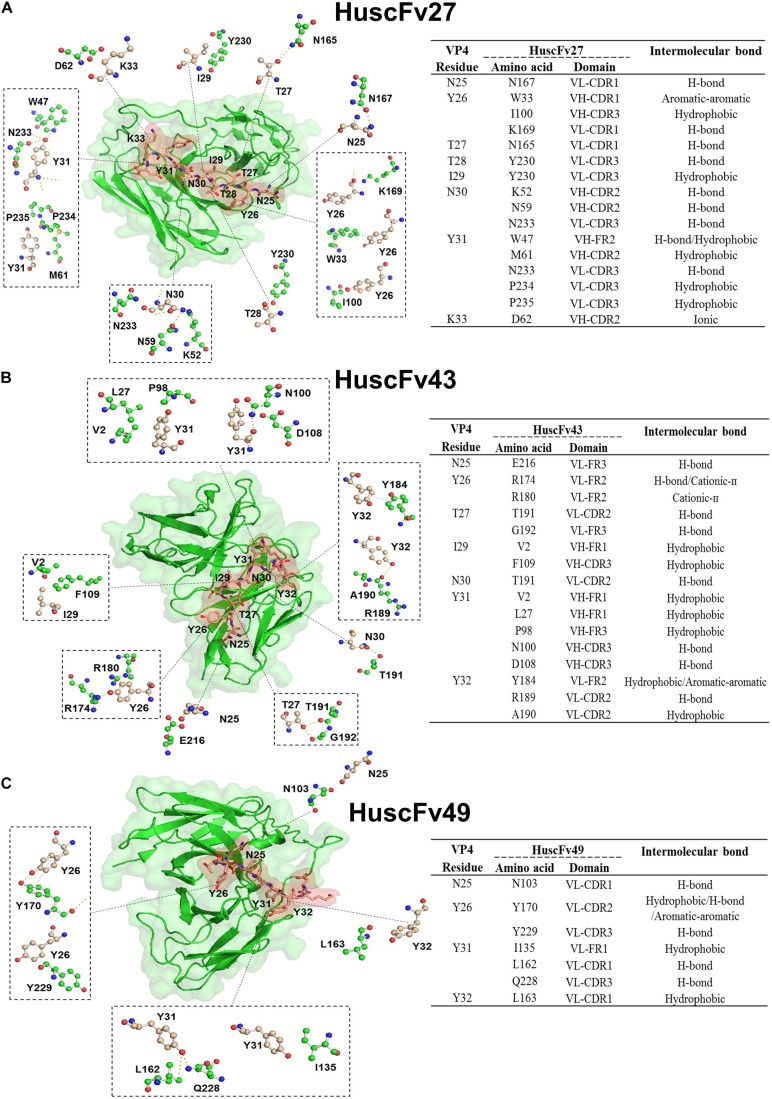
Computerized simulation showing presumptive residues of the overlapped region of peptides 5 and 6 of EV71-VP4 that interacted with the VP4 specific-antibodies. **(A)** HuscFv27, **(B)** HuscFv43, and **(C)** HuscFv49. Tables at the right of the respective figures show details of the VP4 residues that were interacted with residues in the antibody domains, and the resulting interactive bonds.

In order to have some insight into why the three antibodies that bound to the same overlapped residues of peptides 5 and 6 had different anti-viral effectiveness, 3D models of HuscFv27, HuscFv43, and HuscFv49 were docked with the VP4 peptide containing overlapped residues of peptides 5 and 6 using the CABS-dock server for protein-peptide docking. The docking results are shown in Figures 9A–C and in the tables located on the right of individual figures. The results revealed that the HuscFv27 used all complementarity determining regions (CDR1-3) of the VH domain, and CDR1 and CDR2 of the VL domain to interact with several residues of the target peptides *via* hydrogen, hydrophobic, aromatic, and ionic bonds (Figure 9A). The HuscFv43 used VH-CDR3 and VL-CDR2, as well as the canonical framework regions (FRs), to interact with the target peptide (Figure 9B), while the HuscFv49 used only the VL domain (VL-CDRs and VL-FR1) to form a contact interface with the VP4 peptide (Figure 9C).

### Ability of VP4 Specific-Antibodies to Inhibit the A-Particle Infectivity

**FIGURE 10:**
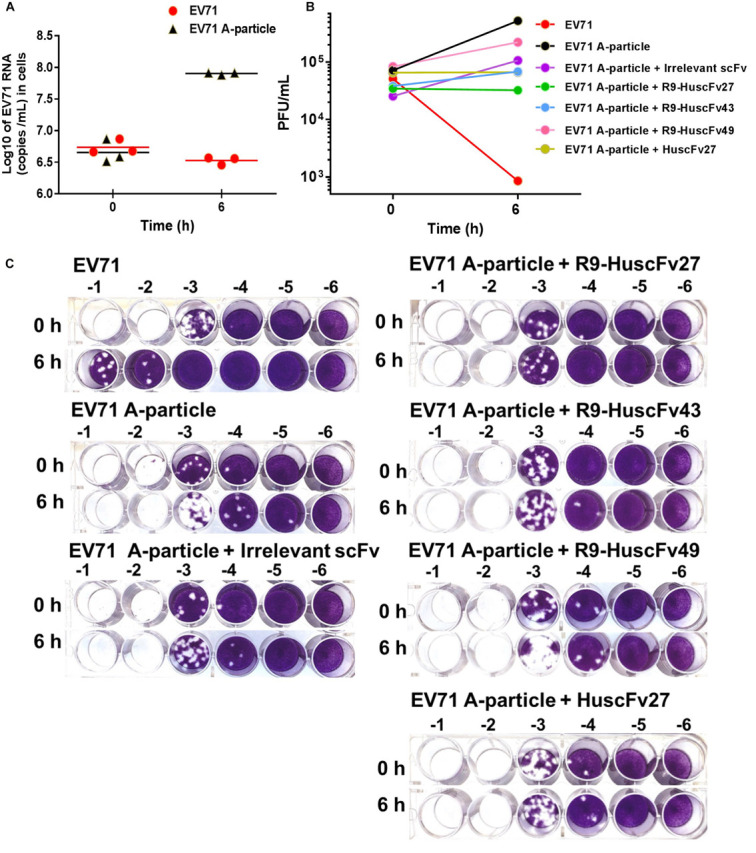
Infectivity of A-particles and wild type EV71, and inhibition of A-particle infectivity by VP4 specific-antibodies. **(A)** Log_10_ of EV71 RNA (copies/mL) recovered from CHO-K1 cells at 0 and 6 h post-infection with A-particles (black triangles) and wild type EV71 (red dots) as determined by qRT-PCR. The A-particles could infect CHO-K1 cells and replicate inside the cells, while the wild type EV71 could not. **(B)** Numbers of infectious particles (PFU/mL) in lysates of CHO-K1 cells added to the mixtures of EV71 A-particles and R9-HuscFv27, R9-HuscFv43, R9-HuscFv49, HuscFv27, and control scFv, compared to CHO-K1 cells infected with A-particles alone and wild type EV71. At 6 h post-infection, the virus RNA increased markedly in the cells infected with A-particles. There was no virus growth in cells added with A-particles mixed with HuscFv27 or R9-HuscFv27. The virus growth in CHO-K1 cells added with a mixture of A-particles and R9-HuscFv43 was less than in the CHO-K1 cells added with a mixture of A-particles and R9-HuscFv49. The control scFv demonstrated some placebo effect compared to the A-particle-infected cells in medium which might be due to steric hindrance of the VP4. The numbers of infectious particles in the lysates of CHO-K1 cells added with wild type EV71 at 6 h post-infection were few. **(C)** The results of plaque assays on RD cells. Plaque assay was performed by diluting the virus-containing samples (culture supernatants and cell lysates) 10-fold serially (-1 to -6 in **C**) in plain DMEM before addition to RD cells in the wells of a 24-well cell culture plate (5 × 10^4^ cells/well).

CHO-K1 cells which are non-permissive to the wild type EV71 can be infected by VP4-externalized A-particles, i.e., the protruded-VP4 on the A-particles could directly make pore on the CHO-K1 plasma membrane for RNA passing out directly to the cytoplasm, regardless of cellular receptor-virus interaction (Curry et al., 1996). Therefore, in order to investigate ability of the VP4 specific-antibodies in inhibiting the virus genome release through interfering with the VP4 membrane activity, the experiments involving CHO-K1 cells infected with A-particles and wild type EV71 were performed and compared. EV71 wild type and A-particles were incubated separately with CHO-K1 cells (MOI 10) for 0 and 6 h. As shown in Figure 10A, the virus RNA copy numbers recovered from the wells incubated with A-particles for 6 h were significantly higher than those from the wells at 0 h, which indicates that the A-particles could mediate release of the virus genome into the cytoplasm and replicate therein. It is should be noted that infection of CHO K1 cells by the A-particles is a single-round infection. The authentic virus progeny released from the A-particle-infected-CHO-K1 cells could not infect new CHO-K1 cells. The virus RNA amounts recovered from wells added with wild type EV71 at 0 and 6 h were not different, which indicates inability of the wild type EV71 to infect the cells, which verified the previous notion.

The 6-h samples of wells containing CHO-K1 cells added with the A-particles in medium alone had the highest increment of the numbers of infectious particles as determined by plaque assay on RD cells (Figures 10B,C). The 6 h samples from wells added with a mixture of A-particles and HuscFv27 or R9-HuscFv27 to CHO-K1 cells revealed no increment of infectious virus particles compared to the 0 h samples, which indicates that the two antibodies could inhibit A-particles infectivity. The effectiveness of R9-HuscFv43 in inhibiting A-particle infectivity was higher than that of R9-HuscFv49, but these two antibodies were both less effective than HuscFv27 and R9-HuscFv27. The control (irrelevant) scFv showed some placebo effect against A-particle infectivity which should be due to steric hindrance than specific binding activity. The 6 h samples from the wells which CHO-K1 cells were added with wild type EV71 had only a small number of infectious particles, which once again supports that the wild type virus could not infect the cells. The results indicate that the VP4 specific-antibodies could inhibit virus replication through interfering with the VP4-membrane pore-forming activity (genome release).

The HuscFv27 and R9-HuscFv27 were the most effective in inhibiting A-particle infectivity on CHO-K1 cells. These antibodies were found to interact presumptively with the VP4 T28, which in Poliovirus is a critical residue for forming ion channel for genome release (Danthi et al., 2003). Moreover, the VP4-T28 hydroxyl group of one capsid protomer formed hydrogen bond with the myristate carbonyl oxygen of another protomer for pentamer stability. Because HuscFv27 and the transbody counterpart could enter the virus-infected cells, their binding to T28 should as well disturb the T28-myristate hydrogen bond between capsid protomers, which should impair the capsid (virus) morphogenesis, hence less virus particles released from the infected cells.

### Effect of VP4 Specific-Antibodies Could Affect Viral Capsid Protein Production

**FIGURE 11:**
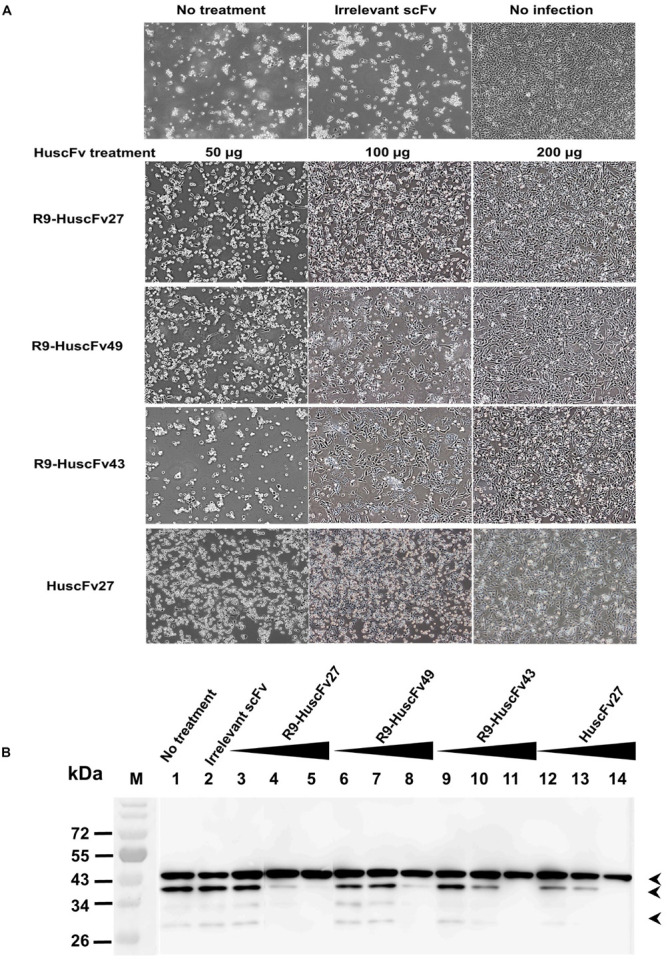
Effects of VP4 specific-antibodies on capsid protein production in EV71-infected cells. RD cells were infected with EV71-C4 (representative) and treated with HuscFvs/R9-HuscFvs (50, 100, and 200 μg or 15, 30, and 60 μg per 5 × 10^5^ RD cells – which were non-cytotoxic doses). Controls were treated with control (irrelevant) scFv or medium. Normal cells were included in the experiments. **(A)** Appearance of the infected cells after various treatment. **(B)** One day after incubation, the content in each flask was subjected to three freeze-thaw cycles. The debris was removed, each lysate was precipitated, and the pellet was mixed with protein sample buffer and subjected to SDS-PAGE and Western blot analysis by probing with mouse anti-VP2 MAb and mouse anti-β-actin MAb. HRP-conjugated mouse IgGκ binding protein and substrate were added, consecutively, with washing between steps. The relative amounts of VP0, VP2, and β-actin were visualized by ECL. M, protein mass markers; lane 1, infected cells in medium; lane 2, infected cells treated with 200 μg of control scFv; lanes 3–5, 6–8, 9–11, and 12–14 were infected cells treated with 50, 100, and 200 μg of R9-HuscFv27, R9-HuscFv49, R9-HuscFv43, and HuscFv27, respectively. Production of VP0 (middle arrowhead) and VP2 (lower arrowhead) in the infected cells treated with VP4 specific-antibodies were reduced in an antibody dose-dependent manner. β-actin (upper arrowhead) was used for normalization.

Because the effective antibodies inhibited the virus replication and genome release, this should bring about less production of intracellular virus proteins. Experiments were performed to determine the amounts of VP0 and VP2 as representatives of the virus structural proteins in the infected cells with and without treatment with VP4 specific-antibodies. Enterovirus-infected RD cells (MOI 1.0) were cultured in medium containing 50, 100, and 200 μg of HuscFv27/R9-HuscFvs for 24 h. Appearance of the infected cells after different treatments are shown in Figure 11A. Thereafter, the cell lysates were subjected to SDS-PAGE and Western blotting by probing the separated proteins with mouse anti-VP2 MAb and mouse anti-β-actin MAb. The amounts of VP0, VP2, and β-actin bands were visualized by enhanced chemiluminescence (ECL). As shown in Figure 11B, production of VP0 and VP2 in infected cells treated with VP4 specific-antibodies was reduced in an antibody dose-dependent manner.

### VP4 Specific-Antibodies Enhanced the Innate Immune Responses of Enterovirus-Infected Cells

**FIGURE 12:**
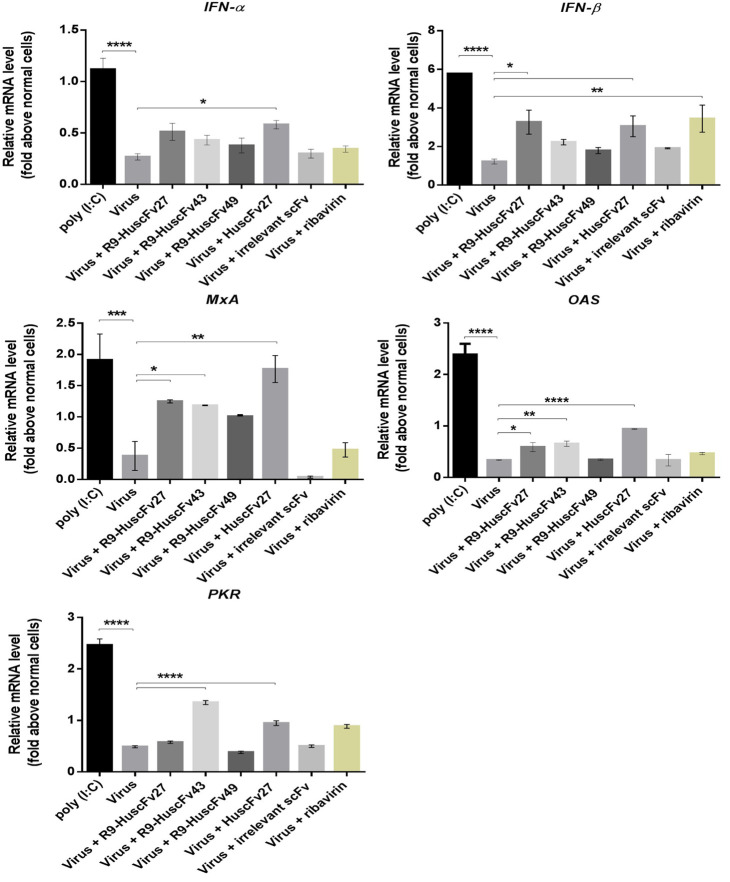
Expressions of the innate immune response genes in EV71-infected RD cells after various treatments. Infected RD cells treated with poly (I:C) (positive control) induced expressions of all of the innate immune response genes tested, including *IFN*-α, *IFN-β, MxA*, *OAS*, and *PKR* many folds above the respective gene expressions in the uninfected (normal) cells (*p* < 0.001), and in the EV71-infected cells (*p* < 0.001). EV71-infected cells treated with HuscFv27 and R9-HuscFv27 had enhanced expressions of *IFN*-β, *MxA*, and *OAS* above those demonstrated by EV71-infected cells (*p* < 0.05). The HuscFv27 also caused significant enhancement of *IFN*-α and *PKR* expressions. The R9-HuscFv43 significantly enhanced *MxA*, *OAS*, and *PKR* expressions, while the R9-HuscFv49 did not have any effect on the innate genes tested compared to infected cells without any treatment or normal cells. The nucleoside analog ribavirin enhanced expressions of *IFN*-β compared to the virus-infected cells (*p* < 0.01), but not the downstream genes of the IFN-IFNRI signaling pathways (*MxA*, *OAS*, and *PKR*). *, *p* < 0.05; **, *p* < 0.01; ***, *p* < 0.001; ****, *p* < 0.0001.

Inhibition of the virus replication should also results in less production of the virus proteins not only capsid proteins, but also non-structural proteins including 2A, 2C, 3C, and 3D which have been known to inhibit the anti-viral innate immunity (please see section “Discussion”). Therefore, experiments were performed to observe whether inhibition of the virus replication by the VP4 specific-antibodies would as well cause consequently restoration of the host innate immune response. Figure 12 shows the results of one of the three independent and reproducible experiments that were performed after the infected cells were treated for 24 h for all genes except *MxA* (as there were no differences in the gene expression at other time points compared to no treatment control). Infected RD cells treated with poly (I:C) (positive control) induced expressions of all of the innate immune response genes tested, including innate interferon genes (*IFN*-α, *IFN*-β) and genes (downstream of the interferon-receptor signaling) coding for innate anti-viral factors (i.e., *MxA*, *OAS*, and *PKR*) many folds above the respective gene expressions in the uninfected (normal) cells (*p* < 0.001), and in the EV71-infected cells (*p* < 0.001). EV71-infected cells treated with HuscFv27 and R9-HuscFv27 had enhanced expressions of *IFN*-β, *MxA*, and *OAS* above those demonstrated by EV71-infected cells (*p* < 0.05). The HuscFv27 also caused significant enhancement of *IFN*-α and *PKR* expressions. The R9-HuscFv43 significantly enhanced *MxA*, *OAS*, and *PKR* expressions, while the R9-HuscFv49 did not have any effect on the innate genes tested compared to infected cells without any treatment or normal cells. The nucleoside analog ribavirin enhanced expressions of *IFN*-β compared to the virus-infected cells (*p* < 0.01), but not the downstream genes of the IFN-IFNRI signaling pathways.

## Discussion

CVA16 and multiple subgenotypes of EV71 are the common causative agents of HFMD outbreaks, while strains of CVA6 serotype took preeminence in a particular year and caused a disease outbreak (Puenpa et al., 2013; Li J. X. et al., 2016). Since 2001, the EV71 subgenotypes B4, B5, C1, C2, C4, and C5 have been circulating and causing HFMD outbreaks among infants in Thailand (Puenpa et al., 2011). During 2011–2018, the majority of HFMD cases in the Kingdom of Thailand were caused by the EV71 subgenotypes B5 and C4 (Puenpa et al., 2019). Nevertheless, the CVA6 serotype was detected in most HFMD cases in the large 2012 outbreak, followed in falling numbers of infected subjects by CVA16 and EV71, respectively (Puenpa et al., 2013). In August 2017, EV71 subgenotype B5 returned as the principal etiologic agent in an HFMD outbreak (Puenpa et al., 2018). The aforementioned HFMD situation emphasizes the need for a vaccine and an antiviral agent that are broadly effective against all of the prevalent genotypes, as well as against the emerging strains of HFMD-causing enteroviruses. Several investigators have explored potential therapeutics against enterovirus infections, including small molecular inhibitors (e.g., pleconaril that binds VP1) (Ledford et al., 2004), ribavirin (nucleoside analog) (Li et al., 2008), rupintrivir (cysteine-protease inhibitor) (Zhang et al., 2010), siRNA to the virus 3D polymerase (Tan et al., 2007), and lactoferrin (Lin et al., 2002). However, these agents were associated with several different limitations, such as rapid clearance and degradation, toxicity, susceptibility to virus-resistant mutations, and requirement for special delivery system (siRNA). Intravenous immune globulin (IVIG) obtained from blood donors with high EV71 neutralizing antibody titers was used with success for passive immunization (treatment) of severe HFMD cases caused by EV71 (Bayry et al., 2004; Cao et al., 2010). However, the supply of that immune serum is limited, and the virus-neutralizing capacity varies from donor-to-donor and batch-to-batch. Moreover, IVIG recipients may be at risk for potential hazardous effects that include exposure to blood-borne pathogen, possibility of antibody-dependent enhancement (ADE) of viral replication and antibody-dependent cell-mediated cytotoxicity (ADCC), and complement-mediated cell lysis (Ri, 1994; Han et al., 2011; Schneider et al., 2017). Mouse monoclonal antibodies (MAbs) directed against the surface-exposed structural proteins of EV71 (VP1, VP2, and VP3) have been generated for use in HFMD treatment (Chang et al., 2010; Liu et al., 2011; Lim et al., 2012; Kiener et al., 2014; Ku et al., 2015; Ng et al., 2015). On the contrary, MAbE18 generated by immunization induced an unfavorable outcome (transformation of the infectious EV71 virions to A-particles) that facilitated viral genome release into the host cytoplasm regardless of the virus receptors (Plevka et al., 2014). The use of mouse MAbs as therapeutic agents faces limitations, including immunogenicity of the foreign isotypes in human recipients, which may cause consequent adverse effects (e.g., immediate anaphylaxis or delayed serum sickness) (Descotes, 2009), although the immunogenicity of these antibodies can be reduced by humanization. Moreover, *in vitro* experiment showed that EV71 infected cells secrete soluble EV71 procapsids that bind neutralizing antibodies and rescues virus infection (Shingler et al., 2015). The passively administered mouse MAbs can participate in the ADE and ADCC (Chames et al., 2009; Acosta and Bartenschlager, 2016).

VP4, which is a myristoylated capsid protein (∼70 amino acids) that lies on the inner surface of the virus, plays a pivotal role in pore formation under low endosomal pH that allows delivery of the viral genome from endosome into the cytoplasm to initiate viral translation and replication. Recombinant VP4 of human rhinovirus formed a multimeric size-selective pore on a liposome that permitted transport of single-stranded RNA through the pore (Panjwani et al., 2014). Mutation of threonine 28 (T28) of poliovirus VP4 prevents genome delivery to the cytoplasm, which confirms the role of enterovirus VP4 in the infection process, and the critical function of the VP4-T28 residue (Danthi et al., 2003). VP4 myristylation formed by a hydrogen bond between mysristate carbonyl oxygen and the VP4-T28 hydroxyl group of the capsid protomers is a signature feature of members of the *Picornarviridae* family (Moscufo and Chow, 1992), except hepatoviruses and parechoviruses (Stanway and Hyypiä, 1999). This intermolecular linking is pivotal for the infection process, proteolytic processing, and viral particle maturation and assembly by maintaining the conformation of the capsid pentamers [links the 5S-protomers (VP0-VP1-VP3) within the 14S-pentamer], which is required for assembly into both 75S-native empty particle and provirion (Kräusslich et al., 1990; Corbic Ramljak et al., 2018). Importantly, VP4 is highly conserved across EV71 subgenotypes (Zhao et al., 2013), and it shares high identity with the VP4 of other enterovirus serotypes (Supplementary Figure 4). Therefore, an anti-viral agent that targets the VP4 should have broad activity against EV71 strains of all subgenotypes and possibly against other enterovirus serotypes.

In this study, engineered small human antibody variable fragments (HuscFvs) that are five times smaller than IgG (to facilitate enhanced tissue penetrating ability), that are human proteins (so there is no or negligible immunogenicity in humans), that are devoid of Fc fragments (so that they cannot cause ADE and ADCC), and that are cell penetrable (by linking the HuscFvs to a cell penetrating peptide, R9; so that they can effortlessly reach intracellular target, i.e., the endosomal or the cytoplasmic VP4– the latter in the form of nascent polyprotein PP, intermediate P1, and/or intermediate VP0, before virus morphogenesis) were generated using phage display technology. As expected, the R9-HuscFv27, R9-HuscFv43, and R9-HuscFv49 readily entered the virus-infected cells, co-localized, and interacted with the intracellular VP4, as determined by sectional confocal microscopy and immunoprecipitation (revealed by ELISA and Western blotting). HuscFv27 without the cell penetrating peptide could not enter the uninfected cells, but it could enter the EV71-infected cells, and it co-localized and interacted with the intracellular VP4. The ability of the HuscFv27 to enter enterovirus-infected cells should be due to the increase in plasma membrane permeability during virus infection, which is a phenomenon that has also been observed in cells being infected with other viruses including HIV-1 (Cloyd and Lynn, 1991), Dengue virus (Talavera et al., 2004), and influenza virus (Frolov et al., 2003; Yodsheewan et al., 2013). Moreover, the presence of an intracellular target is likely to further promote the cellular entry of the HuscFvs.

From titration using various amounts of HuscFvs and R9-HuscFvs, the antibodies to VP4 at 60 μg or less were not toxic to the RD cells, which were used to represent human cells in the cytotoxic assay. In addition, all of the antibodies could bind to virally produced-VP4 of not only the EV71 subgenotypes, but also other enterovirus serotypes that were tested, including CVA16. These findings motivated us to test the ability of these antibodies to inhibit replication of different EV71 subgenotypes and the closely related CVA16 and CVA6, which are now more often observed as the preeminent causative organisms in HFMD outbreaks.

All of the VP4 specific-antibodies generated in this study were effective in reducing the viral RNA in both the infected cells and in the culture supernatants, as well as reduction of the infectious virions of all enteroviruses that were tested, as determined by plaque assay. They also caused marked reduction of the amounts of VP0 and VP2 in virus-infected cells in an antibody dose-dependent manner. It should be noted that all antibodies bound to an overlapped region of the VP4 peptides 5 and 6 (i.e., 25NYTTINYYKD34), which is located in the N-terminal portion of the VP4. The VP4 N-terminus (residues 1–45) of Rhinovirus (also a member of genus *Enterovirus*) was shown to be as active in membrane permeability/activity in forming size selective pore required for RNA transferring as full-length VP4; and more active than the VP4 C-terminus and the VP1 N-terminus (Panjwani et al., 2016). Another study has shown that sera against the VP4 N-terminus (residues 1–30), but not to the C-terminus, neutralized several Rhinovirus serotypes infectivity (Katpally et al., 2009). Antibodies directed against the VP4 of poliovirus serotype 1 demonstrated neutralizing activity; however, that activity disappeared after the antiserum was incubated with synthetic VP4 (Li et al., 1994). Mice immunized with chimeric VLPs containing 20 N-terminal amino acids of VP4 elicited neutralizing antibodies against EV71 of different genotypes (Zhao et al., 2013). These annotated data not only verify that the VP4 N-terminal portion is important for enterovirus infectivity, they also help to explain the mechanisms that cause the HuscFvs and R9-HuscFvs to inhibit replication and production of infectious particles in the enteroviruses tested. The HuscFvs and R9-HuscFvs are likely to interfere with VP4-mediated genome release, which leads to reduced virus translation and replication (reduction of virus RNA and intracellular virus proteins) in the antibody-treated infected cells. Experiments on the antibody-mediated inhibition of A-particle infectivity verified this speculation. Both HuscFvs and R9-HuscFvs in the cell/virus milieu could enter endosome with the virus, where they can block the VP4-membrane activity therein leading to retention of the virus genome in the endosome Besides, the R9-HuscFvs that enter the cell *via* the plasma membrane penetration can also enter the endosome containing the endocytosed virus and block the genome release. Thus, there are chances that the antibodies could reach the intra-endosomal as well as intracytoplasmic targets.

Virus RNA recovered from the wells to which CHO-K1 cells were added with wild type EV71 were not different at 0 and 6 h incubation, which indicates that the detected RNA at both time points were from the virus particles that attached to the cell exterior and/or remained attached non-specifically to the culture well surface (could not be removed readily by washing). On the contrary, the amount of virus RNA from wells containing CHO-K1 cells infected with A-particles at 6 h incubation was significantly higher than at 0 h, which indicates that the A-particles could infect the CHO-K1 cells and replicate intracellularly. This finding is consistent with the previous finding that the EV71 A-particles, not the wild type virus, could infect CHO cells and replicate within them (Curry et al., 1996). It should be noted here that infection of the CHO-K1 cells by the A-particles is a single-round infection; the so-produced authentic virus progeny cannot infect new CHO-K1 cells. The numbers of infectious particles from A-particle-infected cells were not increased when the VP4-exposed A-particles were pre-incubated with HuscFv27 or R9-HuscFv27 before addition to CHO-K1 cells as determined by plaque forming assay on RD cells. This is in contrast to the cells infected with A-particles in medium alone or added with control antibody, which indicates that the two VP4 specific-antibodies blocked membrane pore-forming activity of the externalized VP4 of the A-particles. The R9-HuscFv43 and R9-HuscFv49 also showed this activity, but to a lesser degree of effectiveness than the HuscFv27 and R9-HuscFv27. These results were also observed from the RNA and plaque recovery assays using RD cells infected with the wild type viruses. Taken together, these results indicate that the VP4 specific-antibodies that were developed in this study inhibit VP4 pore-forming activity, which leads to reduced virus production in the cells and the released infectious virus particles.

In most instances, the HuscFv27 showed the highest effectiveness in reducing the amounts of virus RNA and infectious virus particles from infected cells and/or spent culture fluids. This might be due to the fact that the HuscFv27 could enter most, if not all, of the virus-infected cells that had increased membrane permeability, while the cell penetrating (R9)-HuscFvs (called super-antibodies by Charles Morgan, president of InNexus Biotechnology of Vancouver, Canada, which has developed super-antibody technology) could enter any cells in the culture wells, and if there was no target, then the super-antibodies leave the cells, enter new cells until they find the target, and then they finally accumulate in the cells containing the target (the super-antibodies could last in the body for up to a month, entering and leaving cells until they find their target)^1^. Therefore, the amounts of the cell penetrating antibodies in the virus-infected cells should be less than the amount of HuscFv27 in the infected cells. This is because the R9-HuscFvs sometimes wasted time finding the target, and some of them might remain behind in the uninfected cells.

It was observed that the CPE of RD cells infected with CVA6 was different from that of RD cells that were infected with EV71-A (BrCr), EV71-B5, EV71-C4, and CVA16, whereas the CPE caused by the EV71-C4 was markedly less extensive than those of the cells infected with other enteroviruses (Supplementary Figure 5). It is not known whether the differences in the CPE appearances and severity would reflect differences in the cell membrane permeability. If these were the cases, then it might explain the variable effectiveness of the HuscFv27 against different enteroviruses due to different ability in entering the cells infected with the respective viruses.

In the VP4 molecule, the hydroxyl side chain of the T28 residue of one capsid protomer forms a hydrogen bond with the myristate carbonyl oxygen of another protomer. This interaction stabilizes the pentamer subunit of the virus capsid, which is necessary for virus assembly/morphogenesis and virion stability (Chow et al., 1987; Moscufo and Chow, 1992). Glycine or lysine mutation of the T28 led to virus lethality, while valine or serine substitution reduces virus infectivity and enhances virus susceptibility to heat and neutralizing antibodies (Moscufo and Chow, 1992; Danthi et al., 2003). Inhibition of enterovirus VP4 myristoylation by 2-hydroxymyristic acid has been shown to specifically inhibit the cleavage of VP0 (Tan et al., 2016). Among the HuscFvs/R9-HuscFvs to VP4 in this study, the HuscFv27 and/or R9-HuscFv27 were the most/highly effective antibodies against the viruses. The two antibodies presumptively interacted with T28 *via* VP4 hydrogen bonding (computerized simulation). In the case where the antibodies that traversed across the plasma membrane and entered cytoplasm of the infected cells happened to bind to the T28, this should cause disturbance of the threonine-myristate hydrogen bond between protomers; hence, causing sterically strained configurations of the capsid pentamer with the consequence of virus morphogenesis impairment. Virus assembly cannot occur if the T28 cannot form intersubunit interactions with residues from other pentameric VP4 proteins, and these intersubunit interactions cannot be formed if the protomers were not assembled into pentamers (Moscufo and Chow, 1992). Binding of VP4 specific-antibodies to intracytoplasmic nascent polyprotein PP, intermediate P1 and intermediate VP0 could also interfere with the further protein processing, capsid maturation and viral morphogenesis. Although the R9-HuscFv43 and R9-HuscFv49 did not show binding to the T28 by computerized simulation, they bind to the VP4 peptides 5 and 6 (similar region to the HuscFv27/R9-HuscFv27), which means that they should also be able to inhibit VP4 membrane activity as well as capsid maturation. The antibody-mediated inhibition of VP4 activity observed in this study seems to depend also on the enterovirus serotype and strain. The R9-HuscFv49, which were the least effective against EV71, seemed to perform better against the CVA viruses, which have a percent identity of the VP4 molecules to the VP4 of the EV71 subgenotypes that is relatively lower (Supplementary Figure 4).

Innate interferons (IFNs) are the first-line host immunity to viral infections (Kawai and Akira, 2006). Usually, molecular signatures of the infecting viruses alarm the intracellular host pathogen recognition receptors (PRRs) including cytosolic RIG-1-like receptors [retinoic acid-inducible gene 1 (RIG-1), melanoma differentiation-associated gene 5 (MDA5)], AIMS-2-like-receptors (IFI16, DAI, or AIM2 itself) and endosomal Toll-like receptors (TLR7, TLR8 and TLR9), to activate various adaptor proteins, i.e., TRIF (Toll-IL-1 receptor domain-containing adaptor inducing IFN-β), Cardif/MAVS, and TRAFs (tumor necrosis factor-associated factors) (Yoneyama et al., 2005; Kuo et al., 2013; Schneider et al., 2014; Zhu et al., 2015). These factors consequently activate kinases (TBK1 and IKKs) leading to phosphorylation and dimerization of interferon regulatory factor 3/7 (IRF3/7), transcription factors for production of innate interferons, including type-I IFN-β and type-III IFN-λ (IL-28/IL-29) (Alexopoulou et al., 2001; Yoneyama et al., 2004; Cui et al., 2014). The IFNs mediate autocrine and paracrine stimulation of the host cells through JAK/STAT signaling pathways leading to activation of many interferon-stimulated genes (ISGs) and production of various anti-viral factors, i.e., 2’, 5’ OAS, PKR, MxA, and ISGs, as well as MHC class I molecules for cytotoxic lymphocyte activation (Cui et al., 2014; Schneider et al., 2014). However, enteroviruses produce several non-structural proteins to subvert the host innate defense factors for their fitness. EV71 uses many non-structural proteins, including 2A proteases (2A^pro^) (Yi et al., 2011; Lu et al., 2012), 2C helicase (Zheng et al., 2011), 3C proteases (3C^pro^), and 3D RNA-dependent RNA polymerase (3D^*pol*^) (Lei et al., 2010; Kuo et al., 2019) to circumvent innate IFNs by inhibiting multiple steps in IFN-induction and the IFN-IFNRI signaling pathway, and antagonizing host innate anti-viral response (Lu et al., 2012; Taylor and Mossman, 2013; Wang et al., 2013, 2017). The EV71-2A^pro^ cleaves multiple sites of mitochondria antiviral signaling (MAVS) proteins (IPS-1, VISA, and Cardif), RIG-I (Wang et al., 2013) and MDA5, and consequently suppresses IRF3 activation (Kuo et al., 2013). Moreover, 2A^pro^ induces degradation and reduction of the level of type I interferon (IFN)-α/β receptor 1 (IFNAR1) by inhibition of IFN-mediated phosphorylation of signal transducers and activators of transcription 1/2 (STAT1/STAT2), Janus activated kinase 1 (JAK1), and tyrosine kinase 2 (TYK2) (Lu et al., 2012; Wang et al., 2015), which inhibits expressions of innate anti-viral factors (e.g., ISG15, ISG56, MxA, Mx1, 2′,5′-OAS, and PKR) (Lu et al., 2012; Zou et al., 2015). In addition, the 2C^pro^ can interact with the IPT domain of RelA (p65) leading to reduced formation of heterodimer p65/p50, which is the active NF-κB form (Du et al., 2015). The EV71-2C helicase inhibits TNF-α-mediated NF-κB activation by suppressing IκB kinase (IKKβ) phosphorylation. EV71-3C^pro^ suppresses RIG-1-mediated innate IFN responses by interacting with the RIG-I-MAVS complex (Lei et al., 2010), and also inhibits the TLR3 signaling pathway by cleaving Q312-S313 and Q189-S190 on TIR-domain-containing adapter-inducing interferon-β (TRIF) and the IFN regulatory factor 7 (IRF7) (Lei et al., 2011, 2013). It suppresses NF-κB activation by cleaving complexes of transforming growth factor-β-activated kinase 1 (TAK1) and TAK1-binding proteins (TABs) (Lei et al., 2014). The EV71-3C^pro^ also blocks JAK1-STAT signaling by cleaving IRF9 (Hung et al., 2011). EV71-3D^*pol*^ inhibited MDA5-mediated beta-interferon (IFN-β) promoter activation (Kuo et al., 2019). Therefore, during EV71 infection, innate IFNs are less detected in cell-based systems and animal model, which resulted in impaired expression of various ISGs to fight against the virus (Zheng et al., 2011). In the EV71-infected RD cells, IFN-α and IFN-β were slightly upregulated. *IFN*-β transcripts were induced robustly in RD cells after stimulation with poly (I:C), but decreased significantly at 24- and 36-h post-infection (Wang et al., 2016). In this study, poly (I:C) also upregulated innate immune response genes. The HuscFvs/R9-HuscFvs to VP4 inhibited genome release and virus morphogenesis; this should result in less production of non-structural proteins/viral enzymes particularly those that inhibit the innate antiviral responses and thus an observed increment in the expressions of innate immune response genes. The degree of effectiveness in innate gene enhancement seemed to be commensurate with the ability of the antibodies to inhibit virus replication and infectious particle generation (i.e., the HuscFv27/R9-HuscFv27 were the most effective followed by the R9-HuscFv43, while the R9-HuscFv49 did not show any effect on the innate gene response that was tested). Ribavirin, is the virus nucleoside analog that was found to enhance *IFN*-β, but not the antiviral factor genes downstream of the innate IFNs-IFNRI signaling pathway. This can be explained by the fact that the ability of the nucleoside analog in virus replication inhibition occurred later than the effect of the VP4 specific-antibodies, which tended to block at the initial step of the virus infection (i.e., genome release and possibly later capsid assembly). Supplementary Figure 6 illustrates potential mechanisms of the VP4 specific-antibodies of this study.

## Conclusion

The human single-chain antibodies directed against VP4 that were generated in this study showed high effectiveness against the EV71 subgenotypes, as well as against heterologous enterovirus serotypes, including CVA16 and CVA6. The effective antibodies should be further investigated for their clinical application as a safe and broadly effective HFMD therapy.

## Data Availability Statement

All datasets presented in this study are included in the article/Supplementary Material.

## Ethics Statement

The animal study was reviewed and approved by the Animal Care and Use Committee of the Faculty of Medicine Siriraj Hospital, Mahidol University, Bangkok, Thailand (COA no. SI-ACUC 008/2561).

## Author Contributions

WC: conceptualization, funding acquisition, resources, project administration, data curation, formal analysis, supervision, validation, visualization, methodology, and writing the manuscript (original draft and final version). SP: investigation, methodology, computerization, statistical calculation, visualization, and assisted WC in manuscript writing and editing. JD: investigation, methodology. WS, ST, JT, and KP: provided supervision and guidance to SP, and methodology. NS: resources, project administration, supervision, and methodology. YP, NO, and RG: virus isolation, propagation, and methodology. All authors contributed to the article and approved the submitted version.

## Conflict of Interest

The authors declare that the research was conducted in the absence of any commercial or financial relationships that could be construed as a potential conflict of interest.

## Footnotes

1 https://www.newscientist.com/article/dn4881-super-antibodies-break-the-cell-barrier/#ixzz6IG LlAZvn

## Supplementary Material

FIGURE S1 Biotin-labeled overlapped peptides of the EV71-VP4 (peptides 1–12, started from the first amino acids of the VP4) that were used in determining the region of VP4 bound by the effective VP4 specific-antibodies. Each peptide contains 15 amino acids with 10 overlapped residues with the adjacent peptide sequences.

FIGURE S2 Infectivity of EV71 A-particles compared to wild type EV71 on CHO-K1 cells and ability of the VP4 specific-antibodies in inhibiting the A-particle infectivity by interfering with membrane-pore forming activity of the VP4. **(A)** CHO-K1 cell monolayer was established in two six-well culture plates. Cells in 3 wells of each plate were added with wild type EV71 (upper row) and A-particles (lower row). Immediately after adding the viruses, the fluids in all wells of plate 1 were discarded; the cells were washed and added with 1 mL of medium followed by TRIzol^®^ reagent for RNA preparation (0 h samples were obtained). Plate 2 was incubated at 37°C in a CO_2_ incubator for 1 h to allow virus entry. Then, fluids in all wells were removed and the cells were washed, replenished with 1 mL of fresh culture medium, and incubated further for 5 h. All wells were then added with TRIzol^®^ reagent (6 h samples were obtained). Virus RNA amounts in the 0 and 6 h samples were quantified by qRT-PCR and compared between wild type and A-particle infections. **(B)** Experiments for determining the ability of the VP4 specific-antibodies to inhibit A-particle infectivity *via* inhibition of membrane pore-forming activity of the VP4, A-particles (with protruded VP4) in medium, or mixed with HuscFvs/R9-HuscFvs/control (irrelevant) scFv, or wild type EV71 in medium were added individually to 3 wells containing 10^6^ CHO-K1 cells in 6-well culture plates. The fluids in all wells of plate 1 were discarded immediately; the wells were then washed, added with 1 mL of fresh culture medium, and the plate was subjected to three freeze-thaw cycles. The cell debris was removed by centrifugation. The 0 h samples were obtained. The remaining plates were incubated for 1 h to allow virus entry; the fluids in all wells were discarded, and the cells were washed, added with 1 mL of fresh culture medium, and incubated further for 5 h. The plates were then subjected to three freeze-thaw cycles, the cell debris in all wells was removed by centrifugation, and the 6 h samples were obtained. The infectious virus titers in the 0 and 6 h samples were determined by plaque assay performed on the RD cells, and the results were compared among all treatments.

FIGURE S3 The comparative results of plaque assay for detecting infectious virus particles (PFU/mL) in the culture supernatants of infected RD cells after treatment with medium alone (no treatment), HuscFv27, R9-HuscFv27, R9-HuscFv43, R9-HuscFv49, irrelevant (control) scFv and ribavirin. In this experiment, monolayer of RD cells (2 × 10^5^ cells in 1 mL complete DMEM) in individual wells of 12-well culture plates were added with enteroviruses (MOI 0.1 for EV71-A BrCr, EV71-C4 and CVA16; MOI 0.02 for EV71-B5; and MOI 0.05 for CVA6), and incubated at 37°C for 1 h. Extracellular viruses were discarded by washing. The infected cells were added with 60 μg of HuscFvs or R9-HuscFvs (4 wells for each treatment) and incubated at 37°C in a 5%CO_2_ atmosphere. The culture supernatants and the cells were collected when the CPE caused by individual viruses were clearly seen and the supernatants were subjected to the plaque assay of which the results are shown in this figure.

FIGURE S4 Multiple alignments of VP4 amino acid sequences of EV71-A, EV71-B1-B5, EV71-C1-5, CVA16, and CVA6.

FIGURE S5 Monolayer of normal RD cells and the CPE of the cells caused by EV71-A (BrCr), EV71-B5, EV71-C4, CVA16, and CVA6.

FIGURE S6 Potential mechanisms of the VP4 specific-antibodies of this study. HusFvs/R9-HuscFvs in the cell/virus milieu could enter the endosome with the endocytosed virus. The R9-HuscFvs in the cytoplasm could enter the endosome. In both instances, the antibodies inhibit membrane-pore forming activity of the externalized VP4 causing viral RNA retention in the endosome; hence, less cytoplasmic RNA with the consequences of low viral protein production, both structural (capsid) and non-structural, such as 2A, 2C, 3C, and 3D which are innate interferon antagonists; hence restoration of the host innate immunity. Moreover, the VP4 specific-antibodies in the cytoplasm could bind to VP4 in the nascent polyprotein PP and intermediate proteins P1 and VP0, and interfere with the protein processing and morphogenesis; hence, less virus release from cells (not shown in the figure).
