# Supplementary figures and images for: Human Antibodies to VP4 Inhibit Replication of Enteroviruses Across Subgenotypes and Serotypes, and Enhance Host Innate Immunity
Source: Front Microbiol. 2020 Sep 25;11:562768. doi: 10.3389/fmicb.2020.562768 (PMC7545151; doi:10.3389/fmicb.2020.562768)

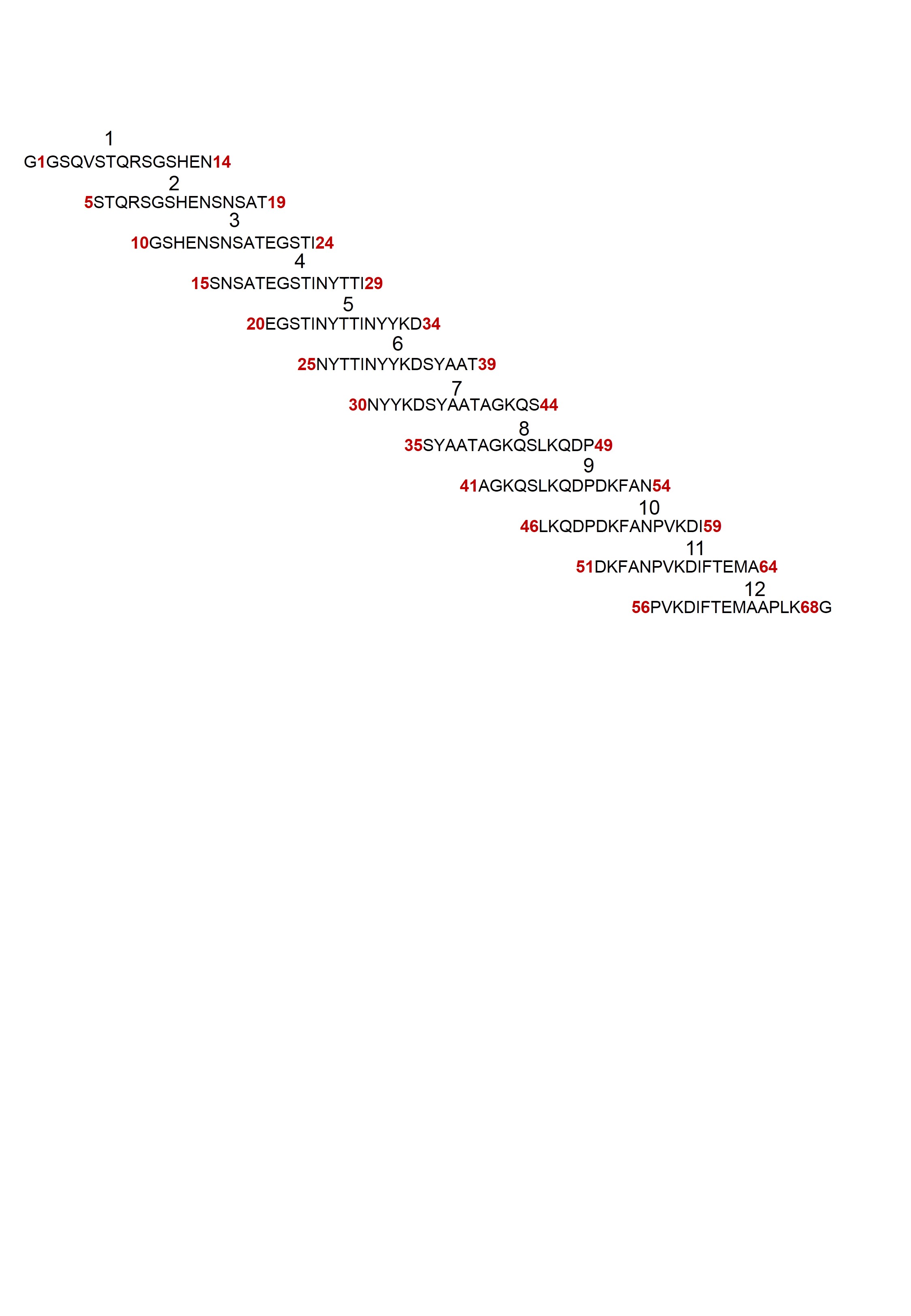

Supplement: FIGURE S1 — Biotin-labeled overlapped peptides of the EV71-VP4 (peptides 1–12, started from the first amino acids of the VP4) that were used in determining the region of VP4 bound by the effective VP4 specific-antibodies. Each peptide contains 15 amino acids with 10 overlapped residues with the adjacent peptide sequences. [file Image_1.JPEG]

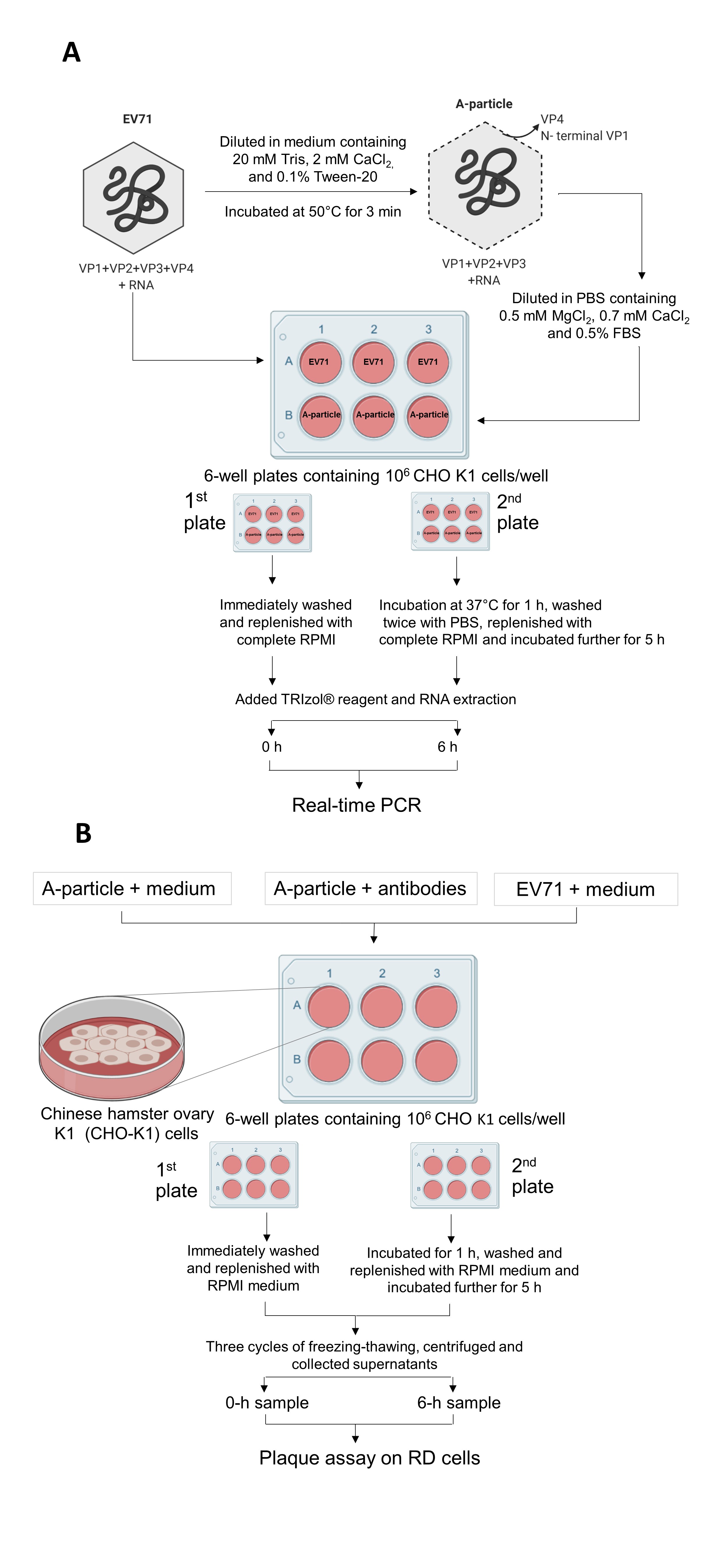

Supplement: FIGURE S2 — Infectivity of EV71 A-particles compared to wild type EV71 on CHO-K1 cells and ability of the VP4 specific-antibodies in inhibiting the A-particle infectivity by interfering with membrane-pore forming activity of the VP4. (A) CHO-K1 cell monolayer was established in two six-well culture plates. Cells in 3 wells of each plate were added with wild type EV71 (upper row) and A-particles (lower row). Immediately after adding the viruses, the fluids in all wells of plate 1 were discarded; the cells were washed and added with 1 mL of medium followed by TRIzol® reagent for RNA preparation (0 h samples were obtained). Plate 2 was incubated at 37°C in a CO2 incubator for 1 h to allow virus entry. Then, fluids in all wells were removed and the cells were washed, replenished with 1 mL of fresh culture medium, and incubated further for 5 h. All wells were then added with TRIzol® reagent (6 h samples were obtained). Virus RNA amounts in the 0 and 6 h samples were quantified by qRT-PCR and compared between wild type and A-particle infections. (B) Experiments for determining the ability of the VP4 specific-antibodies to inhibit A-particle infectivity via inhibition of membrane pore-forming activity of the VP4, A-particles (with protruded VP4) in medium, or mixed with HuscFvs/R9-HuscFvs/control (irrelevant) scFv, or wild type EV71 in medium were added individually to 3 wells containing 106 CHO-K1 cells in 6-well culture plates. The fluids in all wells of plate 1 were discarded immediately; the wells were then washed, added with 1 mL of fresh culture medium, and the plate was subjected to three freeze-thaw cycles. The cell debris was removed by centrifugation. The 0 h samples were obtained. The remaining plates were incubated for 1 h to allow virus entry; the fluids in all wells were discarded, and the cells were washed, added with 1 mL of fresh culture medium, and incubated further for 5 h. The plates were then subjected to three freeze-thaw cycles, the cell debris in all wells was [file Image_2.jpg]

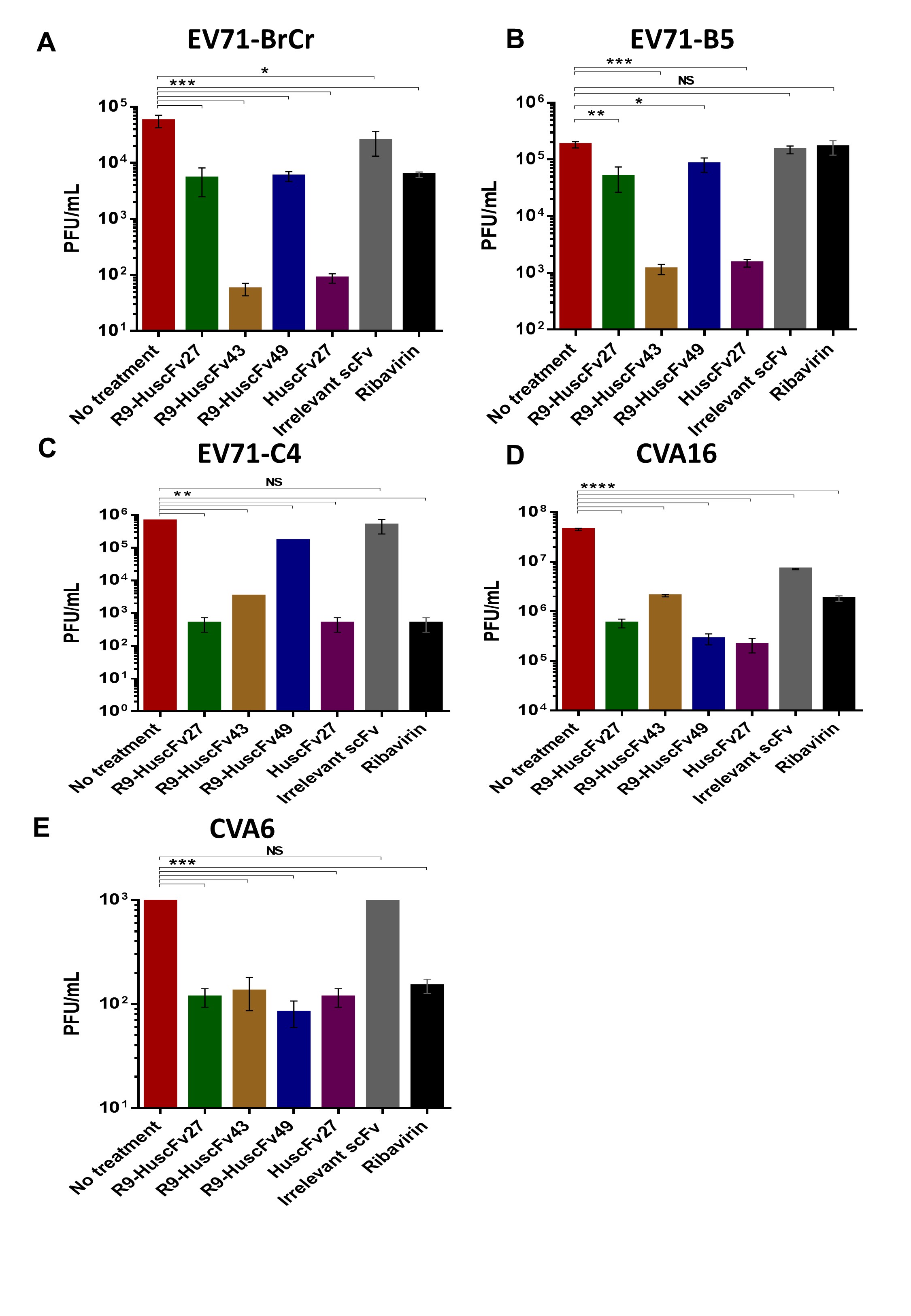

Supplement: FIGURE S3 — The comparative results of plaque assay for detecting infectious virus particles (PFU/mL) in the culture supernatants of infected RD cells after treatment with medium alone (no treatment), HuscFv27, R9-HuscFv27, R9-HuscFv43, R9-HuscFv49, irrelevant (control) scFv and ribavirin. In this experiment, monolayer of RD cells (2 × 10^5^ cells in 1 mL complete DMEM) in individual wells of 12-well culture plates were added with enteroviruses (MOI 0.1 for EV71-A BrCr, EV71-C4 and CVA16; MOI 0.02 for EV71-B5; and MOI 0.05 for CVA6), and incubated at 37°C for 1 h. Extracellular viruses were discarded by washing. The infected cells were added with 60 μg of HuscFvs or R9-HuscFvs (4 wells for each treatment) and incubated at 37°C in a 5%CO2 atmosphere. The culture supernatants and the cells were collected when the CPE caused by individual viruses were clearly seen and the supernatants were subjected to the plaque assay of which the results are shown in this figure. [file Image_3.JPEG]

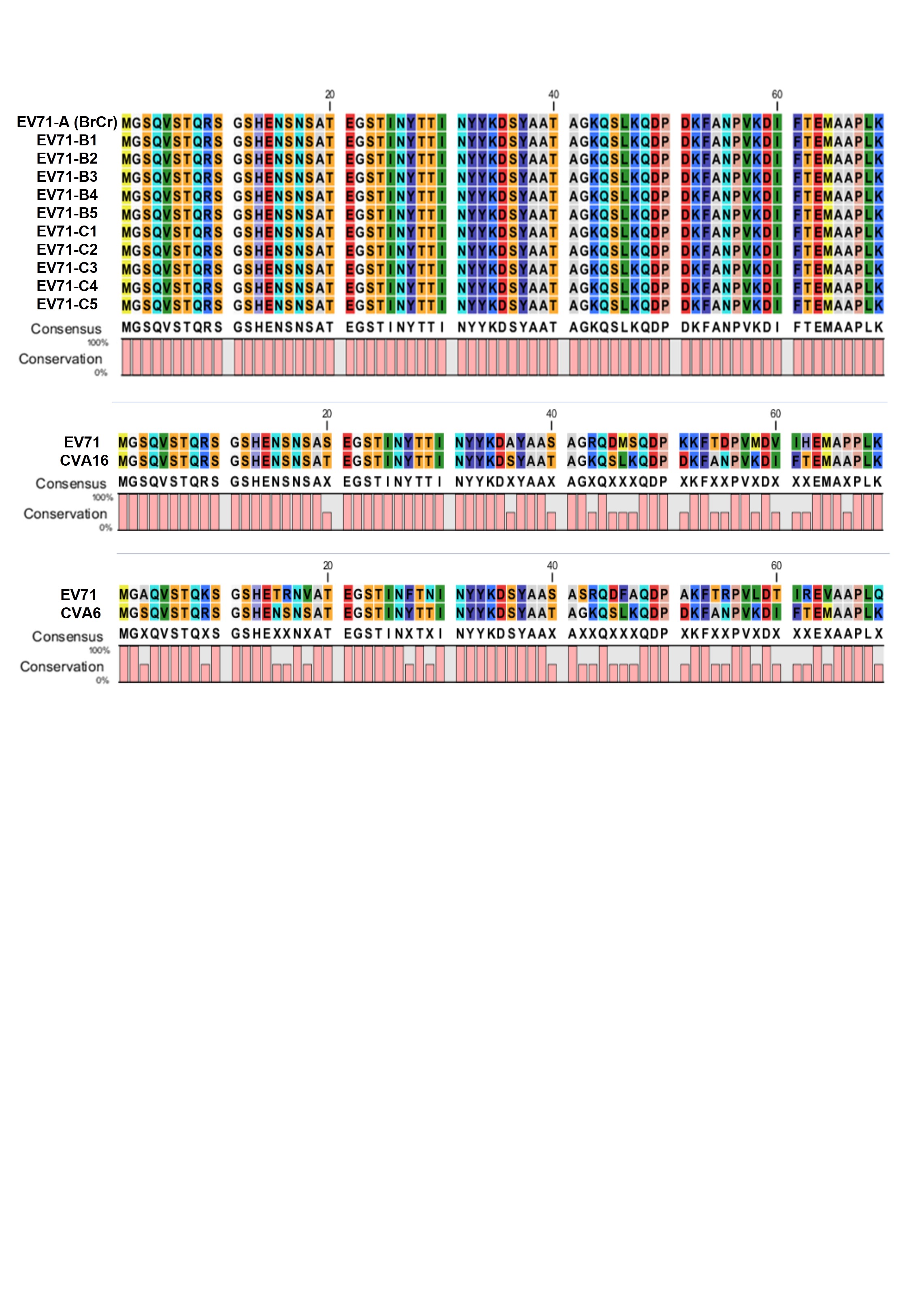

Supplement: FIGURE S4 — Multiple alignments of VP4 amino acid sequences of EV71-A, EV71-B1-B5, EV71-C1-5, CVA16, and CVA6. [file Image_4.JPEG]

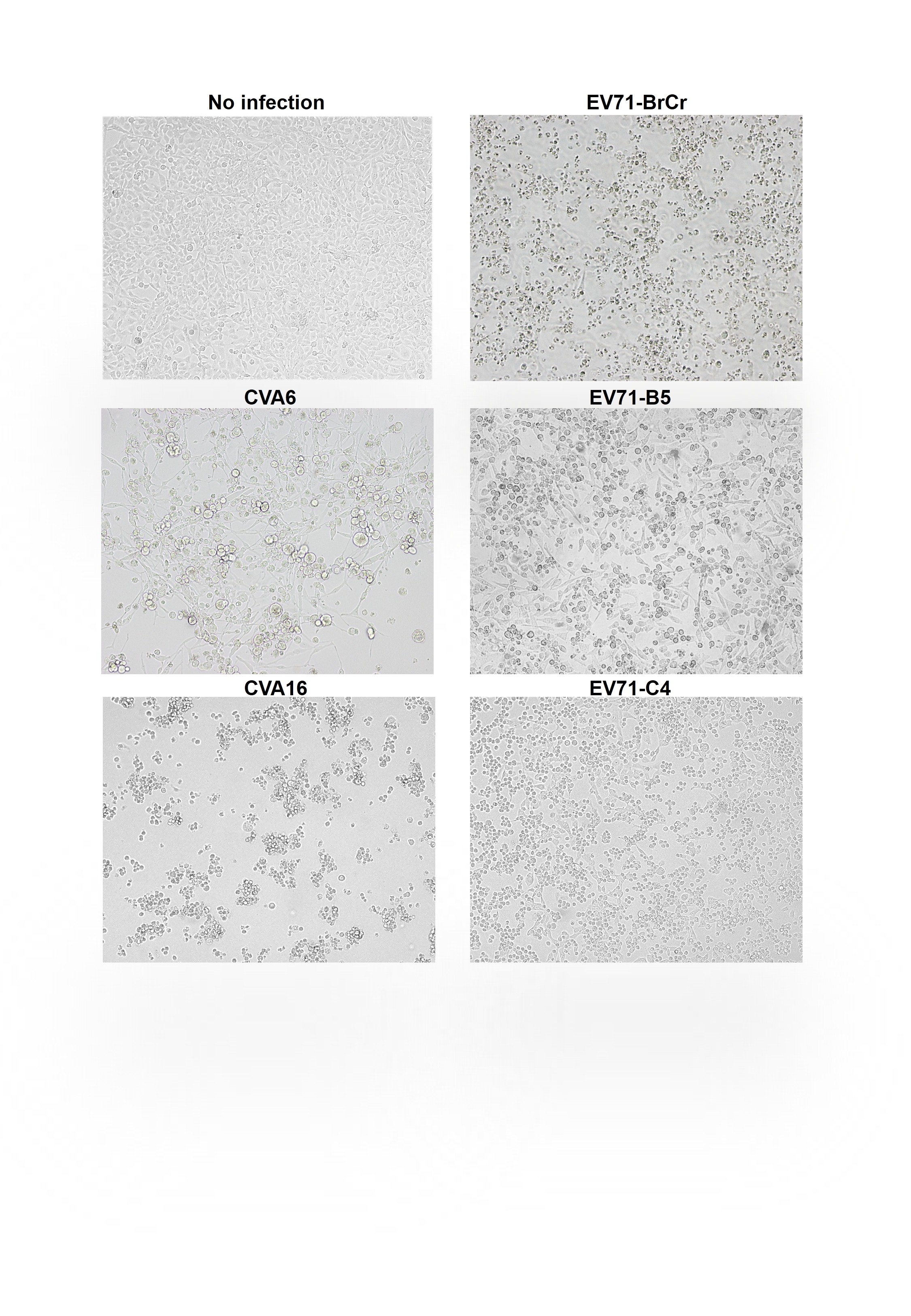

Supplement: FIGURE S5 — Monolayer of normal RD cells and the CPE of the cells caused by EV71-A (BrCr), EV71-B5, EV71-C4, CVA16, and CVA6. [file Image_5.JPEG]

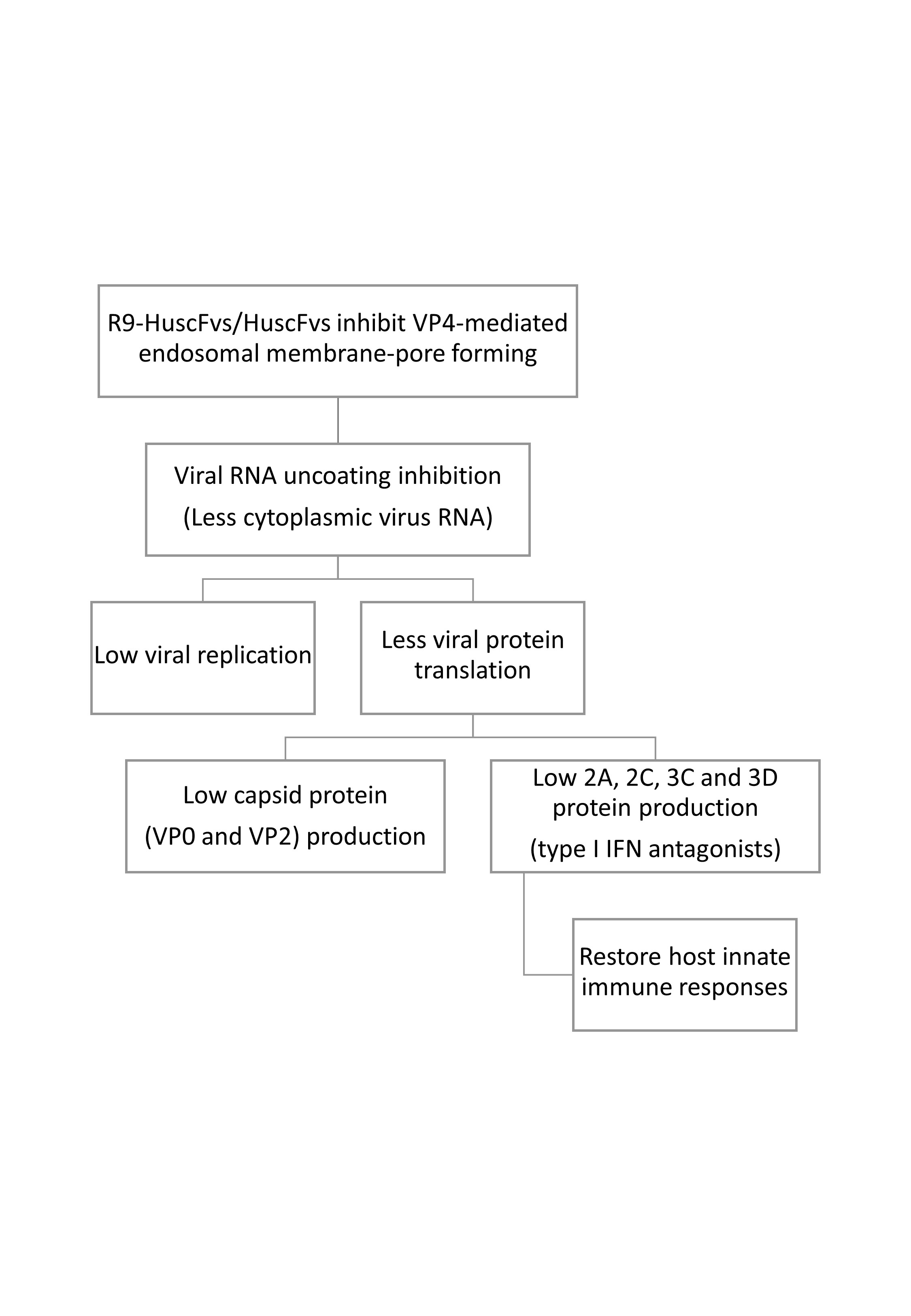

Supplement: FIGURE S6 — Potential mechanisms of the VP4 specific-antibodies of this study. HusFvs/R9-HuscFvs in the cell/virus milieu could enter the endosome with the endocytosed virus. The R9-HuscFvs in the cytoplasm could enter the endosome. In both instances, the antibodies inhibit membrane-pore forming activity of the externalized VP4 causing viral RNA retention in the endosome; hence, less cytoplasmic RNA with the consequences of low viral protein production, both structural (capsid) and non-structural, such as 2A, 2C, 3C, and 3D which are innate interferon antagonists; hence restoration of the host innate immunity. Moreover, the VP4 specific-antibodies in the cytoplasm could bind to VP4 in the nascent polyprotein PP and intermediate proteins P1 and VP0, and interfere with the protein processing and morphogenesis; hence, less virus release from cells (not shown in the figure). [file Image_6.jpg]
