# Supplementary material for: Human Antibodies to VP4 Inhibit Replication of Enteroviruses Across Subgenotypes and Serotypes, and Enhance Host Innate Immunity
Source: Front Microbiol. 2020 Sep 25;11:562768. doi: 10.3389/fmicb.2020.562768 (PMC7545151; doi:10.3389/fmicb.2020.562768)
Supplement: Supplementary file 7 [file Data_Sheet_1.docx]

**Supplementary Material 1**

## Determination of cell culture infectious dose 50 (CCID_50_) of the viruses

The cell culture infectious dose 50 (CCID_50_) of each virus stock was determined according to the protocol described in the fourth edition of the Manual for the Virological Investigation of Polio, World Health Organization, 2004. Briefly, the virus stock was serially diluted 10-fold in plain DMEM, and then added to the wells of 96-well plates. RD cells were added to the virus-containing wells (2 × 10^4^ cells/well) and incubated at 37°C in a 5% CO_2_ atmosphere until the CPE was clearly observed. The Kärber formula was used to calculate the virus CCID_50_ (10^x^/0.1 mL) for each viral stock.

## Supplementary Material 2

## Production of recombinant VP4

For production of recombinant VP4, the consensus EV71-VP4 coding sequence was obtained by multiple alignments of all VP4 sequences of EV71 subgenotypes retrieved from the National Center for Biotechnology Information (NCBI; Bethesda, MD, USA). The pUC57 plasmid containing inserted gene coding for full-length EV71-VP4 consensus sequence with 5′ *Eco*RI and 3′ *Hin*dIII restriction sites was synthesized (GenScript, Piscataway, NJ, USA). The VP4 gene was excised from the recombinant pUC57 plasmid, gel-purified using an extraction kit (Geneaid Biotech, New Taipei City, Taiwan), cloned into the pET-23b(+) expression vector, and the recombinant vector was introduced into JM109 *E. coli*. After verification by DNA sequencing, the plasmids were extracted from the JM109 *E. coli* and transformed into BL21 (DE3) *E. coli*. Appropriately transformed-BL21 (DE3) *E. coli* was grown in Luria-Bertani (LB) broth containing 100 μg/mL ampicillin (LB-A) under induction with 0.5 mM isopropyl β-D-1-thiogalactopyranoside (IPTG) (Thermo Fisher Scientific). The bacterial cells were harvested, lysed with lysis buffer (phosphate buffer, pH 6.2 containing lysozyme, 5 mM EDTA, 50 mM NaCl), sonicated in ice-bath (0.5 cycle, 40% amplitude for 5 min), centrifuged (8000× *g*, 4°C, 30 min) and the pellet was resuspended in buffer (20 mM Tris-HCl containing 8 M urea, 20 mM imidazole, 0.5 M NaCl). The preparation was re-sonicated, filtered through 0.45 μm membrane and applied to a HisTrap™ HP Prepacked Column (GE Healthcare) connected to ÄKTA primeplus system. The protein was allowed to bind to the column until the UV curve peak on the Prime-View monitor was stable. The column was washed three times with: 1) Wash buffer 1 (50 mM Tris-HCl containing 4 M urea, 40 mM imidazole, 0.1% Tritox X-100) until the UV peak was stable; 2) Wash buffer 2 (50 mM Tris-HCl containing 4 M urea, 1% Triton-X-114); and 3) Wash buffer 3 (50 mM Tris-HCl containing 60% isopropanol), respectively. The 6× His-tagged-recombinant protein that bound to the HisTrap column was refolded on the column by adding 20 mM Tris-HCl, 20 mM imidazole, 0.5 M NaCl. Then, the protein was eluted with buffer, pH 9.6 (20 mM Tris-HCl,; 500 mM imidazole and 0.5 M NaCl) (the pH 9.6 kept the recombinant protein in solution). Purity of the eluted recombinant protein was determined by sodium dodecyl sulfate-polyacrylamide gel electrophoresis (SDS-PAGE), Coomassie Brilliant Blue G-250 (CBB) staining, and Western blot analysis by probing the SDS-PAGE-separated preparation with mouse anti-6× His-tag antibody (Bio-Rad Laboratories, Hercules, CA, USA). The purified recombinant 6× His-tagged-protein was verified to be the EV71-VP4 protein by LC-MS/MS.

**Overexpression of VP4 in mammalian cells**

For production of mammalian cell overexpressed-VP4, the VP4 gene was subcloned from the recombinant pUC57 plasmid into a modified pCI-neo Mammalian Expression Vector (Promega Corporation, Madison, WI, USA) containing Flag-tag DNA (Seesuay et al., 2018) and transformed into JM109 *E. coli*. The transformants were screened by polymerase chain reaction (PCR), and the plasmids they contained were verified by DNA sequencing. The pCI-neo plasmids with VP4 coding sequence were transformed into unmethylated JM110 *E. coli*. The plasmids containing *VP4* were extracted from a transformed *E. coli* clone using an EndoFree Maxi Plasmid Kit (Tiangen Biotech, Beijing, China). HEK293T cells were transfected with 25 µg of plasmid preparation using Xfect Single Shots (Maxi) Transfection Reagent (Takara Bio, Shiga, Japan). Two days post-transfection, spent medium was removed from the culture well, and the cells were rinsed twice with PBS and lysed with 1 mL of M-PER Mammalian Protein Extraction Reagent (Thermo Fisher Scientific) supplemented with 1:200 dilution of protease inhibitor cocktail (set-III/EDTA-free) (Calbiochem, Merck KGaA, Darmstadt, Germany) and 25 units/mL Benzonase® Nuclease (Novagen, Merck KGaA, Darmstadt, Germany). The preparation was kept at 25°C until the cells were completely lysed, and then the solution was centrifuged at 15,000× *g* at 4°C for 5 min. The cell lysates were verified for the presence of Flag-tagged fusion proteins by enzyme-linked immunosorbent assay (ELISA) using HRP-conjugated mouse anti-6× His antibody and ABTS substrate, and by Western blot analysis using mouse anti-Flag MAb (Sigma-Aldrich, St. Louis, MO, USA) as the primary antibody, and HRP-conjugated goat anti-mouse isotype (SouthernBiotech, Birmingham, AL, USA) as the secondary antibody. The antigen-antibody reactive bands were visualized by enhanced chemiluminescence (ECL) (LuminataTM Crescendo Western HRP substrate, Millipore, Billerica, MA, USA).

## Supplementary Material 3

## Production of mouse anti-VP4 polyclonal antibodies (PAb)

Animal experiments were approved by the Animal Care and Use Committee of Faculty of Medicine Siriraj Hospital, Mahidol University, Bangkok, Thailand (COA no. SI-ACUP 008/2561). Four-week-old female BALB/c mice (4 mice) were individually and intramuscularly immunized with 20 µg of purified rVP4 mixed with alum adjuvant (1:2) (Thermo Fisher Scientific). Five booster doses of the same immunogen were given at two-week intervals. One week after the last booster, the mice were bled and their serum antibody titers were determined by indirect ELISA against 1 µg of the homologous antigen per microplate well. Immunoglobulins were isolated from the immune sera by 50% ammonium sulfate precipitation, and polyclonal IgG (PAb) was purified using Protein-G Sepharose™ 4 Fast Flow resin (GE Healthcare).

## Supplementary Material 4

## Plaque assay

Plaque assay was performed as previously described (Van der Hoek et al., 2017) with some modifications. Virus preparations serially diluted 10-fold (-1 to -6) in plain DMEM (300 μL) were added to individual wells of a 24-well cell culture plate containing RD cells (5 × 10^4^ cells). After allowing viral adsorption for 1 h, the extracellular viruses were removed. The cells were then rinsed twice with PBS and overlaid with 500 μL of plaque medium [1.5% carboxymethyl cellulose (CMC) (Sigma-Aldrich) dissolved in DMEM]. After incubation at 37°C for 7-10 days, cells were fixed with 10% formaldehyde for 1 h, washed with water, stained with 0.4% crystal violet in 10% ethyl alcohol, kept at RT for 15 min, and then washed. The numbers of well-isolated plaques in individual wells were counted.

**Supplementary Material 5**

## Quantitative reverse transcription-PCR (qRT-PCR)

The qRT-PCR was performed on 300 ng of each RNA preparation using Brilliant II SYBR Green Master Mix Kit, 1 Step (Agilent Technologies, Santa Clara, CA, USA). EV71 and CVA16 RNA were amplified using VP1-specific primers (sense primer: 5′-ATGGKTATGYWAAYTGGGACAT-3′ and anti-sense primer: 5′-CCTGACRTGYTTMATCCTCAT-3′) (Puenpa et al., 2011). The qRT-PCR was 42°C, 30 min; 55°C, 30 min; initial denaturation at 95°C, 10 min; and, 40 cycles of 95°C for 1 min (denaturation), 55°C for 1 min (primer annealing), 72°C for 1 min (extension), and a final extension at 72°C for 10 min. The CVA6 genome was detected using pan-enterovirus primers (sense primer: 5′-CAAGCACTTCTG TTTCCCCGG-3′ and anti-sense primer: 5′-ATTGTCACCATAAGCAGCCA3′). The qRT-PCR was 42°C, 30 min; 55°C, 30 min; initial denaturation at 94°C, 3 min; and, 40 cycles of 94°C for 30 seconds (s) (denaturation), 60°C for 1 min (primer annealing), 72°C for 30 s (extension), and a final extension at 72°C for 7 min. A thermal profile for dissociation curve analysis was set at 95°C for 1 min, then decreased to 60°C for 45 s, and then increased to 95°C for 30 s. A standard curve was constructed from the threshold cycle (Ct) of ten-fold serial dilutions of plasmid carrying the respective genes. The Ct value of each sample was expressed as log_10_ of the viral RNA (copy number/mL) calculated from the standard curve.

**References**

Puenpa, J., Theamboonlers, A., Korkong, S., Linsuwanon, P., Thongmee, C.,

Chatproedprai, S., et al. (2011). Molecular characterization and complete Q16

genome analysis of human enterovirus 71 and coxsackievirus A16 from

children with hand, foot and mouth disease in Thailand during 2008-2011.

*Arch. Virol.* 156, 2007–2013. doi: 10.1007/s00705-011-1098-5

Seesuay, W., Jittavisutthikul, S., Sae-lim, N., Sookrung, N., Sakolvaree, Y., and

Chaicumpa, W. (2018). Human transbodies that interfere with the functions of

Ebola virus VP35 protein in genome replication and transcription and innate

immune antagonism. *Emerg. Microbes Infect.* 7, 41. doi: 10.1038/s41426-018-

0031-3

Van der Hoek, K. H., Eyre, N. S., Shue, B., Khantisitthiporn, O., Glab-Ampi, K.,

Carr, J. M., et al. (2017). Viperin is an important host restriction factor in

control of Zika virus infection. *Sci. Rep.* 7, 4475. doi: 10.1038/s41598-017-04

138-1
